# Stool microbiota variations along the adenoma-colorectal carcinoma sequence — robustness of disease-associated microbial features

**DOI:** 10.3389/fmicb.2026.1822941

**Published:** 2026-08-20

**Authors:** Ana Carvalho, Silvia Gschwendtner, Michael Schloter, Ana M. Gil, Catarina R. Marques

**Affiliations:** 1 Centre for Environmental and Marine Studies (CESAM), Department of Biology, University of Aveiro, Aveiro, Portugal; 2 CICECO-Aveiro Institute of Materials, Department of Chemistry, University of Aveiro, Aveiro, Portugal; 3 Helmholtz Zentrum München, Research Unit for Comparative Microbiome Analysis, Oberschleißheim, Germany

**Keywords:** colorectal cancer, covariate effects, differential abundance analyses, gut microbiome, microbial signatures

## Abstract

**Introduction:**

Colorectal cancer (CRC) has been linked with gut microbiota dysbiosis, thereby fostering the discovery of disease-associated microbial signatures. However, the use of covariate–adjusted methods accounting for confounders effects on microbiome shifts is far from being a gold standard approach, leading to spurious microbial-disease associations. This study aimed to characterize stool microbiota alterations along the adenoma-carcinoma sequence in a Portuguese cohort, and assess the robustness of candidate microbial features.

**Methods:**

Stool samples from healthy individuals (HC), adenoma patients (AP), and CRC patients at early (SI/II) and advanced (SIII/IV) stages, were analyzed using 16S rRNA gene amplicon sequencing. Microbial features’ profiles were compared with publicly available datasets from France, Ireland, Italy, and USA/Canada. Differential abundance (DA) analysis was conducted through multiple DA models (ANCOM-BC2, fastANCOM, MaAsLin2, limma voom, and LEfSe) and statistical approaches, including covariate (age, sex, and body mass index (BMI)) adjustment, prevalence filtering, and analysis of matched subsets.

**Results and discussion:**

Among the altered taxa along de adenoma-carcinoma sequence, *Sutterella* and *Desulfovibrio* were generally enriched across different DA tools and matched subset analyses, while *Parasutterella* and *Adlercreutzia* were depleted. *Sutterella*, *Parasutterella*, and *Adlercreutzia*, were already altered in adenoma and early-stage CRC samples, maintaining or increasing their effect sizes towards advanced CRC stages. This pattern highlights the potential of these taxa as early microbial signatures of colorectal cancer progression. In contrast, *Fusobacterium*, which is a widely accepted CRC biomarker, was not detected after covariate adjustment. Cross-cohort comparison revealed a limited reproducibility of microbial features (i.e., *Dialister* was enriched in the Portuguese and French cohorts; *Parasutterella* and *Terrisporobacter* were depleted in the Portuguese and USA/Canada datasets), being most of them cohort-specific.

**Conclusion:**

Overall, our results strengthen the importance of covariate control towards the identification of potentially robust and reproducible microbial markers of CRC onset and progression.

## 1 Introduction

Colorectal cancer (CRC) is one of the most prevalent cancers worldwide, with the third highest incidence and second highest mortality rates across all cancers (Ferlay et al., 2024). It is a multifactorial disease, with the majority of cases arising from spontaneous mutations (60–65%), highlighting the importance of modifiable risk factors such as diet, lifestyle, and environmental exposures in CRC development. Among these, the gut microbiota has increasingly been associated with CRC initiation and progression (Zhang et al., 2023; White and Sears, 2024).

A healthy gut microbial community contributes to host homeostasis by facilitating the digestion of complex dietary components and producing beneficial metabolites that modulate immune responses and maintain intestinal integrity. In contrast, alterations in microbial composition, namely characterized by reduced abundance of probiotic bacteria, proliferation of opportunistic pathogens, and loss of diversity, can lead to dysbiosis which impairs intestinal homeostasis and triggers disease development, including CRC (Kumar et al., 2023).

High-throughput metabarcoding approaches based on the sequencing of marker genes have enabled detailed profiling of gut microbial communities and the identification of candidate microbial markers for early detection of CRC (Wang et al., 2023). Stool-based microbiome biomarkers are particularly attractive due to their non-invasive nature and potential to complement current CRC screening tools (e.g., colonoscopy, stool-based tests). Moreover, specific bacterial taxa have been associated with CRC pathogenesis through inflammatory and/or genotoxic mechanisms (Kumar et al., 2023). For instance, genotoxic *Escherichia coli* (e.g., colibactin- and/or cytolethal distending toxin-producing) strains have been described as potential drivers of carcinogenesis (Wang and Fu, 2023). *Fusobacterium nucleatum* has been largely proposed as a CRC biomarker due to its enrichment in advanced stages of CRC (Yamamoto et al., 2021) and opportunistic behavior (Kostic et al., 2012), and has been shown to promote carcinogenesis via activation of Wnt/β-catenin and NF-κB signaling pathways (Kostic et al., 2013; Rubinstein et al., 2019). Other taxa, such as *Parvimonas micra* (Yachida et al., 2019), *Desulfovibrio* (Yachida et al., 2019; Uchino et al., 2021), and *Prevotella* (Du et al., 2022) have also been linked to CRC carcinogenesis; while it has been reported the depletion of beneficial bacteria like *Bifidobacterium* (Thomas et al., 2019; Zhang et al., 2023), *Ruminococcus* (Feng et al., 2015), and *Anaerobutyricum hallii* (formerly *Eubacterium hallii*) (Du et al., 2022).

Despite these associations, the identification of universal microbial markers for CRC has proven challenging. Discrepancies across studies often arise from the use of distinct methodologies and techniques, including differences in sample processing, DNA extraction methods, primer selection, targeted hypervariable regions of the 16S rRNA gene, sequencing platforms, and bioinformatic pipelines (Órdenes et al., 2024). Moreover, substantial inter-individual variability in microbiome composition further complicates the discovery of representative biomarkers (Villéger et al., 2018). Host genetics, age, sex, body mass index (BMI), and lifestyle are also major determinants of gut microbiome structure and can confound disease associations if not properly accounted for (Tito et al., 2024).

Currently, there is no consensual statistical approach to control covariates in microbiome analyses (Thomas et al., 2019; Wirbel et al., 2024). This is due to the compositional nature of microbiome sequencing data, where relative abundances are interdependent (Gloor et al., 2017). However, the use of covariate-adjusted differential abundance (DA) models and complementary sensitivity analyses has been shown to boost the specificity and robustness of microbiome associations with disease status and stages, decreasing false-positive rates and enabling more accurate detection of biologically meaningful microbial features (Lin and Peddada, 2024; Wirbel et al., 2024).

In parallel, geographic and population-level factors are increasingly recognized as relevant drivers of microbial variation (Maghini et al., 2025). Differences in diet, lifestyle, geoclimatic conditions, and environmental exposures can modulate both the healthy and disease-associated microbiomes, therefore constraining the reproducibility of microbial signatures linked to CRC across populations. Despite this, although Portugal presents one of the highest CRC incidence rates in Europe, ranking 6th in incidence and 12th in mortality (Ferlay et al., 2024; Elmadani et al., 2025), the stool microbiota of Portuguese CRC patients is still underexplored. According to the Portuguese National Cancer Registry, colon cancer was the second most incident cancer in 2021, and rectal cancer ranked sixth (Registo Oncológico Nacional [RON], 2024). Yet, microbiome-based studies in this population are scarce.

In this study, we hypothesized that CRC onset and progression is associated with shifts in the stool microbiota of the Portuguese cohort, which remain robust after constraining the analyses with confounding factors. To this end, we firstly aimed to characterize the stool microbiome of a cross-sectional Portuguese cohort, comprising healthy individuals, adenoma patients, and CRC patients (across stages SI/II and SIII/IV), using 16S rRNA gene amplicon sequencing. Secondly, we intended to identify differentially abundant microbial taxa that could be promising candidates to flag out CRC onset and progression, through the application of unadjusted and covariate-adjusted methods, which were complemented by sensitivity analyses to assess the influence of DA models and statistical approaches applied, as well as potential confounding variables (i.e., age, sex and BMI) on microbiome variation. We have also hypothesized that, while a subset of these microbial features are shared across populations, a number of signatures are cohort-specific. To test this hypothesis, we evaluated the reproducibility of candidate microbial features across populations, by comparing the Portuguese cohort with publicly available datasets from cohorts in France, Ireland, Italy, and USA/Canada, accounting for demographic, geographic and methodological differences.

A major novelty and contribution of this study rely on the fact that, to the best of our knowledge, this is the first Portuguese CRC microbiome study integrating stage-resolved microbiota profiling with systematic robustness assessment across multiple DA frameworks accounting for biases from confounder covariates, and independent datasets.

## 2 Materials and methods

### 2.1 Ethical statement and sample collection

The present work started in 2014 with the collaboration of the local Hospital Unidade Local de Saúde da Região de Aveiro, E.P.E. (formerly Centro Hospitalar do Baixo Vouga, E.P.E.), and was approved beforehand by the Ethics Committee (no. 056950) and the Administration Council of the Hospital. The study was conducted in accordance with the Helsinki Declaration. A written Informed Consent Form and a questionnaire (to get data related to sex, age, and body mass index-BMI) were obtained from all participants. Recruited subjects were divided into three clinical groups: healthy individuals with no previous or current diagnosis of CRC or other diseases (HC), adenoma patients (AP), and CRC patients (CRC) (Table 1). CRC patients were further down-grouped according to disease staging, as assessed by the Hospital pathologist, based on the TNM (Tumor, Nodes, and Metastases) classification system (Rosen and Sapra, 2023): Stage I/II (SII), and Stage III/IV (SIII/IV). Healthy individuals of the HC group met the following inclusion criteria: over 18 years old of either sex; with no diagnosed illnesses, particularly gastrointestinal; no consumption of antibiotics within a 6-month prior to sampling; consented to volunteer stool samples. For AP and CRC groups were included participants over 18 years old of either sex; diagnosed with altered intestinal mucosa, colon cancer, or rectal cancer (pathological stage available according to TMN classification for CRC patients); without combined (e.g., diabetes) or previous malignancies; without antibiotic consumption in the past 3 months; without impaired consciousness of mental disease. Patients regularly taking other specific medicines (i.e., nonsteroidal anti-inflammatory drugs, metformin, probiotics, or statins) or previously subjected to surgical interventions, chemo- or radiotherapy treatments, were not included in the study. For each volunteered participant were collected the following data: age, sex, weight, and height, being the two latter used to compute the body mass index (BMI).

**TABLE 1:** Demographic information of participants in the Portuguese cross-sectional cohort.

	Clinical groups		
Characteristics	HC	AP	CRC
	(*n* = 17)	(*n* = 9)	(*n* = 24)
**Sex (*n*)**			
Male	3	4	16
Female	14	5	8
Age (mean ± SD)	37.9 ± 11.9	69.7 ± 11.0	72.6 ± 10.8
BMI (mean ± SD)	23.9 ± 4.0	29.1 ± 2.6	26.1 ± 4.4
Cancer stage (n)			
SI/II	-	-	14
SIII/IV	-	-	10

The table details the number of individuals (n) stratified by sex in each clinical group, by CRC staging for CRC patients, while age and BMI are represented as mean ± standard deviation per clinical group. HC, healthy controls; AP, adenoma patients; CRC, colorectal cancer patients (stages SI/II and SIII/IV); BMI, body mass index; SD, standard deviation.

Sterile stool collection kits with detailed instructions were provided to all volunteers. Immediately after reception, the stool samples were aliquoted under sterile conditions, frozen in liquid nitrogen, and stored at -80°C until further analysis.

### 2.2 Microbial DNA extraction

Microbial DNA was extracted from stool samples using the QIAmp^®^ PowerFecal^®^ DNA Kit (Qiagen, Germany), following manufacturer’s instructions. DNA concentration and purity were assessed using NanoDrop spectrophotometer, and subsequently stored at -20°C until further processing. In order to monitor for potential contamination, blank extraction controls were included in DNA extraction. A commercially-available mock community standard (HM-783D BEI Resources, NIAID, NIH, United States) was processed alongside samples as a positive control to validate DNA extraction.

### 2.3 16 S rRNA gene amplification and sequencing

The V3-V4 hypervariable region of the 16S rRNA gene was amplified using the universal eubacterial primers 347F (5′-GGAGGCAGCAGTRRGGAAT-3′) and 803R (5′-CTACCRGGGTATCTAATCC-3′) (Nossa et al., 2010). The PCR mixture contained 100 ng DNA template, 12.5 μL NEBNext high fidelity polymerase (New England Biolabs, Ipswich, United States), and 3 pmol of each primer, for a total volume of 25 μL. To ensure reproducibility, negative and positive (with the mock community sample HM-783D, BEI resources) extraction controls, a negative amplification control (no DNA template), and a mock community sample (1:100 diluted) spiked into a stool sample, were all included in the amplification. The PCR run was as follows: 30 s at 98 °C; 19 cycles of 10 s at 98 °C, 30 s at 60 °C, 30 s at 72 °C; and 5 min at 72 °C. The amplicons from triplicate PCR runs were verified in an electrophoresis agarose gel, pooled together, and purified using AMPure XP beads (Beckman Coulter Life Sciences, Indianapolis, United States). The purified amplicons were quantified in a Fragment Analyzer (Advanced Analytical, Ankeny, United States) by applying the Agilent DNF-473 NGS Fragment Analysis Kit (1–6,000 bp). Library preparation was conducted through indexing, using the Nextera XT Index Kit v2 (Illumina, Inc. San Diego, CA, United States). A total of 25 μL of reaction contained 10 ng of DNA template, 12.5 μL of 2 × NEBNext High Fidelity Polymerase, and 2.5 μL of each indexing primer. The PCR conditions were: 30 s at 98°C; 8 cycles of 10 s at 98°C, 30 s at 55°C, 30 s at 72°C; 5 min 72°C. After the verification of the index PCR (iPCR) amplicons in an electrophoresis agarose gel, they were diluted to 4 nM, pooled and sequenced on an Illumina MiSeq instrument using the MiSeq Reagent Kit v3 (600 Cycle) (Illumina, San Diego, CA, United States), according to the manufacturer’s guidelines.

### 2.4 Data processing and statistical analyses

Raw FASTQ sequences were demultiplexed and stripped of the index and adapter sequences using the MiSeq sequencer software. Data quality filtering was performed in QIIME2 through the ARGUS computational platform (Bolyen et al., 2019). Primer sequences were trimmed from the sequences using the DADA2 plugin (Callahan et al., 2016), with the following parameters: 20 bp removed at each end of the amplicons, reads were truncated at 280 (forward primer) and 220 (reverse primer) positions, with expected error of 5 (forward) and 6 (reverse), respectively. Amplicon sequence variants (ASVs) were annotated using the SILVA database, version 138.1, release 99% (Quast et al., 2013). The taxonomy table, ASV table, and metadata were exported from QIIME2 and imported into RStudio (R version 4.4.2) (R Core Team, 2024), to perform data analyses. Singletons and ASVs with at least 10 reads in the DNA extraction negative control and in the PCR no template control were removed to exclude potential contamination, resulting in an average read loss of 2.59%. For validating the sequencing methodology, mock communities and negative controls were characterized (Supplementary Figure S1), and rarefaction curves indicated that the sequencing depth was sufficient for progressing with further analyses (Supplementary Figure S2).

All statistical analyses were performed in R (v4.4.2). Alpha diversity was calculated with phyloseq, assessing microbiome diversity through Shannon, Simpson, and Inverse Simpson, and species richness via the Observed index (McMurdie and Holmes, 2013). Alpha diversity indices were assessed for normality through Shapiro-Wilk test and for homogeneity of variances with Levene’s test. A one-way analysis of variance (ANOVA) was performed where normality requirements were met, otherwise, a non-parametric Kruskal-Wallis test was used. Beta diversity analyses were performed on a Bray-Curtis dissimilarity matrix (vegan package) (Oksanen et al., 2024) from ASV counts normalized into relative abundance, with 999 permutations. Beta diversity was first visualized as a Principal Coordinates Analysis (PCoA) plot by clinical group. Permutational multivariate analysis of variance (PERMANOVA) was performed alongside beta diversity to assess microbial community differences using the adonis2 function. Univariate and multivariate PERMANOVA analyses were performed to evaluate the effect of each variable and potential confounding factor (clinical group, age group, sex, BMI) on the community structure. Furthermore, distance-based redundancy analysis (dbRDA) was applied to determine the contribution of each variable on the microbial community (capscale function). Finally, beta dispersion analysis (via betadisper function) was carried out to assess homogeneity of multivariate dispersion among groups. All multivariate analyses accounted for the potentially confounding covariates in the collected metadata. Specifically, as categorical variables, we considered sex (female and male) and age group (age classes by decade, from 20–29 to 80–89 year-olds); and BMI was included as a numerical variable. The *p*-values were adjusted for multiple testing (*q*-value) using Benjamini-Hochberg false discovery rate (FDR, significance with *q* < 0.05).

Taxonomy-level assessment of the microbial community composition per clinical group was carried out by calculating taxa relative abundance at the phylum, family, and genus level, expressed as average percentages (%). Venn diagrams were constructed to visualize shared and unique ASVs among clinical groups, as well as shared and unique core microbiota within each clinical group; core microbiota was defined by a > 75% prevalence threshold. The figures were created using R packages pheatmap (Kolde, 2025) or ggplot2 (Wickham, 2016).

### 2.5 DA robustness assessment and sensitivity analyses

To evaluate the robustness of disease-associated microbial community differences, sensitivity analyses of confounding covariates were performed, by comparing DA methods adjusted versus unadjusted for covariates (i.e., age group, sex, BMI); different DA models; non-filtered versus filtered datasets (by a 10%-prevalence filter); multiple correction for false positives; matched subsets to reduce effect size of potentially confounding variables. Matched subsets of the Portuguese cohort were created for sensitivity analyses (Supplementary Table S1). Due to the disperse distribution of age groups and sex between clinical groups, age-matched cohort was composed of subjects aged 35–70 years old (*n* = 25), and sex-matched cohort was created by matching number of male and female subjects by group as closely as possible, filtering out outlier aged subjects when possible (*n* = 36). BMI-matched cohort was comprised of subjects with overlapping BMI (*n* = 26).

DA models herein used (cf. Table 2) included ANCOM-BC2 (Analysis of Compositions of Microbiomes with Bias Correction 2) (Lin and Peddada, 2024), fastANCOM (Zhou et al., 2022), MaAsLin2 (Microbiome Multivariable Associations with Linear Models 2) (Mallick et al., 2021), limma voom (Law et al., 2014; Ritchie et al., 2015), and LEfSe (Linear Discriminant Analysis (LDA) Effect Size) (Cao et al., 2022). All models were applied to the global dataset, whereas LEfSe was not tested on matched subgroups due to poor statistical performance.

**TABLE 2:** General characteristics of the differential abundance (DA) methodologies applied in this study, as well as testing options considered.

DA models	Uses	Compositional data	Covariate adjustment	Input data	Grouped at genus level	Data normalization and transformation	Prevalence filtering	Statistics
ANCOM-BC2	Microbiome data	Yes	Adjusted	Absolute abundances	Yes	Log-transformed ratios	0 and 10%	⋅ Wald test ⋅ Multiple testing correction by BH
	Microbiome data	Yes	Non-adjusted	Absolute abundances	Yes			
fastANCOM	Microbiome data	Yes	Adjusted	Absolute abundances	Yes	Log-transformed ratios	0 and 10%	⋅ Linear model, *t-*test ⋅ Multiple testing correction by BH
	Microbiome data	Yes	Non-adjusted	Absolute abundances	Yes			
MaAsLin2	Microbiome data	No	Adjusted	Absolute abundances	Yes	CLR	0 and 10%	⋅ Linear model, *t*-test ⋅ Multiple testing correction by BH
	Microbiome data	No	Non-adjusted	Absolute abundances	Yes			
Limma voom	RNA-seq	No	Adjusted	Absolute abundances	Yes	TMM normalization and limma-voom transformation	0 and 10%	⋅ Moderated *t*-test from limma’s empirical Bayes model ⋅ Multiple testing correction by BH
	RNA-seq	No	Non-adjusted	Absolute abundances	Yes			
LEfSe	Microbiome data	No	Non-adjusted	Absolute abundances	No	CPM	0%	⋅ Kruskal-Wallis test ⋅ Multiple testing correction by BH (microbiomeMarker implementation)

BH, Benjamini-Hochberg statistical method to control False Discovery Rate (FDR); CLR, Centered Log-Ratio; CPM, Counts Per Million; TMM, trimmed mean of M-values.

For ANCOM-BC2 global, pattern, and pairwise analyses were performed using the ancombc package (Lin and Peddada, 2024), resulting in a log fold change (LFC) value for each comparison that reflects the difference in bias-corrected abundances between groups. The global test is analogous to ANOVA, with HC as reference group. To complement the global analysis, multiple pairwise comparisons were calculated among all groups, while controlling the mixed directional false discovery rate (mdFDR). The pattern analysis identified monotonically increasing or decreasing trends along AP and CRC staging, with HC as reference group.

fastANCOM analysis was carried out per the fastANCOM R package (Zhou et al., 2022), using the default parameters. fastANCOM estimates log fold changes between groups while accounting for compositional bias. Taxa were considered differentially abundant according to the fastANCOM rejection criteria (REJECT = TRUE), reflecting significance after compositional bias correction.

MaAsLin2 was performed through the MaAsLin2 package (Mallick et al., 2021). Microbial abundance data were normalized using centered log-ratio (CLR) transformation to account for compositionality of microbiome sequencing data. MaAsLin2 fits an independent linear model for each microbial feature to estimate regression coefficients. Associations with *q* < 0.25 were considered significant, consistent with the exploratory framework commonly adopted by microbiome association studies.

Limma voom was carried out using limma and edgeR packages (Ritchie et al., 2015; Chen et al., 2025). Library size normalization was performed using the standard trimmed mean of M-values (TMM) normalization, followed by voom transformation (Law et al., 2014) to convert normalized counts to log2 counts-per-million and generate precision weights suitable for linear model. Linear models were fitted for each genus, followed by Bayes moderation of standard errors.

LEfSe analysis was performed (Segata et al., 2011) using microbiomeMarker package (Cao et al., 2022). Significant features were defined as LDA score > 3 and *p* < 0.05 with non-parametric Kruskal-Wallis test.

Overall, genera were considered significantly altered when *p* < 0.05. All methods implemented *p*-value adjustment for mixed directional false discovery rate by Benjamini-Hochberg method (*q* < 0.05 threshold for significance, except MaAsLin2). Taxa evidencing a consistent association with clinical groups throughout the distinct methods applied were deemed robust microbial features and promising candidates as CRC biomarkers.

### 2.6 Cross-cohort analysis of publicly available stool 16S rRNA datasets

Microbial features identified for the Portuguese cohort were compared with previously published stool 16S rRNA gene amplicon sequencing datasets from healthy individuals and CRC patients. Selection criteria for datasets were as follows: analysis performed on stool samples of healthy controls versus CRC patients (AP group not mandatory); cohort should be of a similar background/lifestyle to the Portuguese population; sequencing performed on the Illumina MiSeq platform targeting the V3 and/or V4 region of the 16S rRNA gene; metadata must include sex and age information for each individual subject. Since the cohort from Italy contained only one AP patient, this group was excluded from this cohort.

Raw sequencing data were retrieved from Sequence Read Archive, European Nucleotide Archive, and mothur project databases. Four publicly available datasets were selected, specifically, cohorts from Ireland (PRJEB50080) (Flemer et al., 2018), Italy (PRJNA356414) (Russo et al., 2018), France (PRJEB6070) (Zeller et al., 2014), and North America (United States/Canada)^1^ (Zackular et al., 2014; Table 3). Together with the Portuguese cohort, the combined dataset included 360 individuals (129 HC, 98 AP, and 133 CRC patients). Raw sequencing data from external cohorts were processed using the same bioinformatics pipeline described above to ensure methodological consistency. Datasets were analyzed in R to characterize alpha and beta diversity across cohorts. Alpha diversity of the merged datasets was calculated as described above for the Portuguese cohort. Beta diversity was assessed by Bray-Curtis dissimilarity, ASVs from the merged dataset were normalized into relative abundances and agglomerated to genus level to account for inter-cohort differences. Univariate and multivariate PERMANOVA models including clinical, country, age group, sex, and BMI covariates (staging not included due to data unavailability for some cohorts) were performed, followed by dbRDA and beta dispersion assessment as aforementioned. Differentially abundant taxa within each cohort were identified using ANCOM-BC2 global analysis as described above, controlling for potential confounding covariates (country of origin, age group, sex, BMI), enabling cross-cohort comparison of microbial signatures.

**TABLE 3:** General characteristics of the cohorts included in the present study: country of origin of the cohort subjects; targeted region of 16S rRNA gene sequencing; number of samples and distribution by clinical group and sex; mean age and body mass index (BMI) of cohort members.

Characteristics	Cohorts			
	Flemer et al. (2018)	Regueira-Iglesias et al. (2023)	Zackular et al. (2014)	Zeller et al. (2014)
Country of origin	Ireland	Italy	United States/Canada	France
Samples (*n*)	73	18	90	129
HC (M/F)	23 (7/16)	9 (6/3)	30 (11/19)	50 (24/26)
AP (M/F)	21 (16/5)	0	30 (18/12)	38 (26/12)
CRC (M/F)	29 (17/12)	9 (4/5)	30 (21/9)	41 (24/17)
Age (mean ± SD)	59.6 ± 12.4	79.2 ± 7.1	58.6 ± 10.7	63.3 ± 9.4
BMI (mean ± SD)	28.3 ± 5.9	25.0 ± 4.0	28.2 ± 5.9	25.4 ± 4.1

Italy cohort contained one AP subject, which was excluded from further analyses. HC, healthy controls; AP, adenoma patients; CRC, colorectal cancer patients; SD, standard deviation; M/F, male/female subjects.

## 3 Results

### 3.1 Characteristics of the Portuguese cross-sectional cohort

Table 1 summarizes the demographic characteristics of the Portuguese cohort. Stool samples were obtained from 50 Portuguese volunteers, including 17 healthy individuals (HC), 9 adenoma patients (AP), and 24 CRC patients (CRC) which were further grouped into stage I/II (SI/II; *n* = 14) and stage III/IV (SIII/IV; *n* = 10) CRC patients. The distribution of sex differed among clinical groups with a predominance of females in HC (ca. 82%) and of males (ca. 67%) in CRC group, whereas the AP group has a balanced representation of individuals of each sex. As expected, AP and CRC participants were on average older (69.7 ± 11.0 and 72.6 ± 10.9 years, respectively) than those in the HC group. BMI also presented subtle variations among clinical groups, being higher in AP (29.1 ± 2.6), followed by CRC (26.1 ± 4.4) and HC (23.9 ± 4.0) groups. Given the observed variations, the demographic and clinical group variables were considered in downstream analyses to account for possible confounding effects.

### 3.2 Characterization of the stool microbiome of the Portuguese cohort

#### 3.2.1 Sequencing output

The sequencing of the 16S rRNA gene V3-V4 region generated a total of 4,190,489 reads for all 50 samples, ranging from 34,839 to 153,787 reads per sample, and an average sample read count of 81,172.36, resulting in 3,803 ASVs. Of these, 3,618 were assigned at the family level and 2,946 at the genus level.

#### 3.2.2 Analysis of microbiome diversity and potential confounders

**FIGURE 1:**
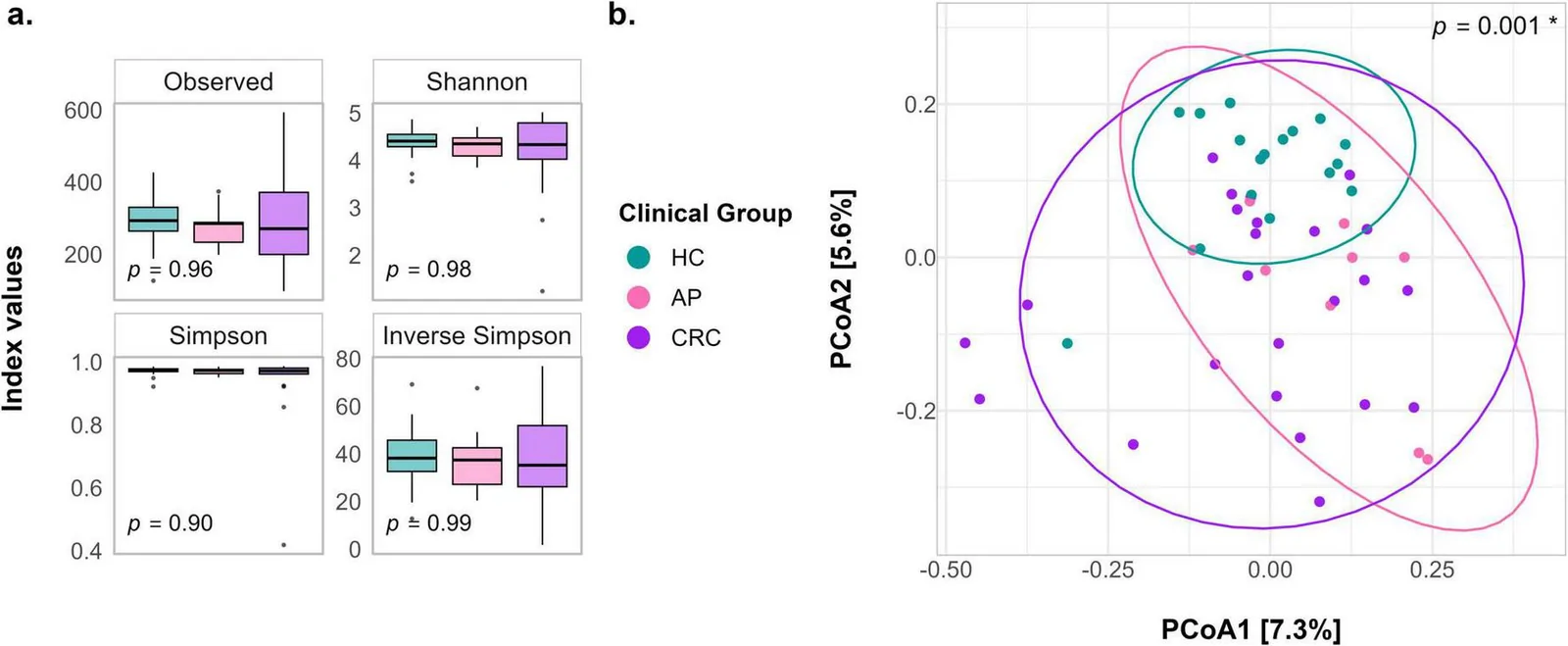
Microbial diversity and community composition in stool samples from the Portuguese cohort. **(a)** Alpha diversity indices (Observed, Shannon, Simpson, Inverse Simpson) plotted across clinical groups, showing no significant variation. **(b)** Beta diversity analysis of stool samples by clinical group calculated through Bray-Curtis dissimilarity and represented in a PCoA plot. HC, healthy controls; AP, adenoma patients; CRC, colorectal cancer patients.

Alpha diversity analyses resulted in non-significant differences between clinical groups for community richness (Observed) or diversity (Shannon, Simpson, Inverse Simpson) indices (*p* > 0.05; Figure 1a and Supplementary Table S2). On the contrary, beta diversity analysis revealed significant variations in the microbial composition between groups. The PCoA ordination based on Bray-Curtis dissimilarity showed partial clustering of HC samples, whereas AP and CRC groups were more dispersed in the plot (Figure 1b). The univariate PERMANOVA identified significant associations between Bray-Curtis beta diversity and the clinical group (composed of HC, AP, CRC-SI/II and CRC-SIII/IV), which explained 7.8% of microbiome variance (*p* < 0.05; Supplementary Table S2). However, in the multivariate PERMANOVA, no significant differences were identified for either variable (*p* = 0.052 for clinical group), suggesting that the differences between groups can be partially influenced by demographic variables. Beta dispersion analysis also indicated a significant heterogeneity within clinical and age groups (Supplementary Table S2), which may have contributed to the non-significant outcome of the multivariate PERMANOVA. Consistent with PERMANOVA results, the microbiome variation explained by the clinical group in the univariate dbRDA (*R*^2^ = 7.8%, *p* = 0.003, *q* = 0.012) was no longer significant after covariate adjustment in the multivariate model (Supplementary Figure S3 and Supplementary Table S2).

#### 3.2.3 Taxonomic and community structure of the stool microbiota

**FIGURE 2:**
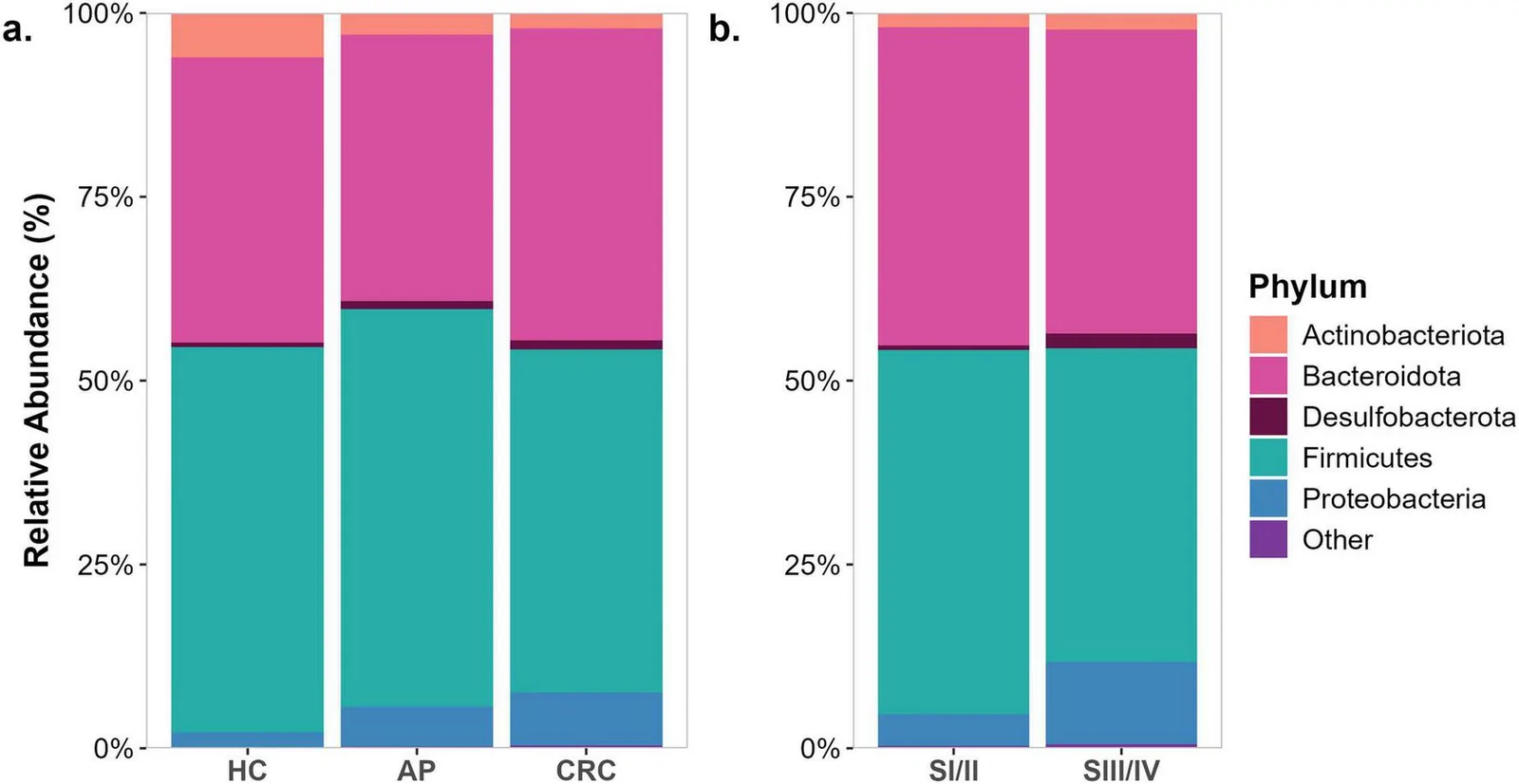
Characterization of stool microbiota communities of the Portuguese cohort at the phylum level. Bar plots represent average relative abundance of phyla by: **(a)** clinical group, comparing HC, AP, and CRC; and by **(b)** CRC staging, comparing SI/II and SIII/IV stages of CRC patients. The “Other” phyla amounted to < 1% of the identified ASVs in all clinical groups. HC, healthy controls; AP, adenoma patients; CRC, colorectal cancer; SI/II and SIII/IV are, respectively, the CRC stages I/II and III/IV.

**FIGURE 3:**
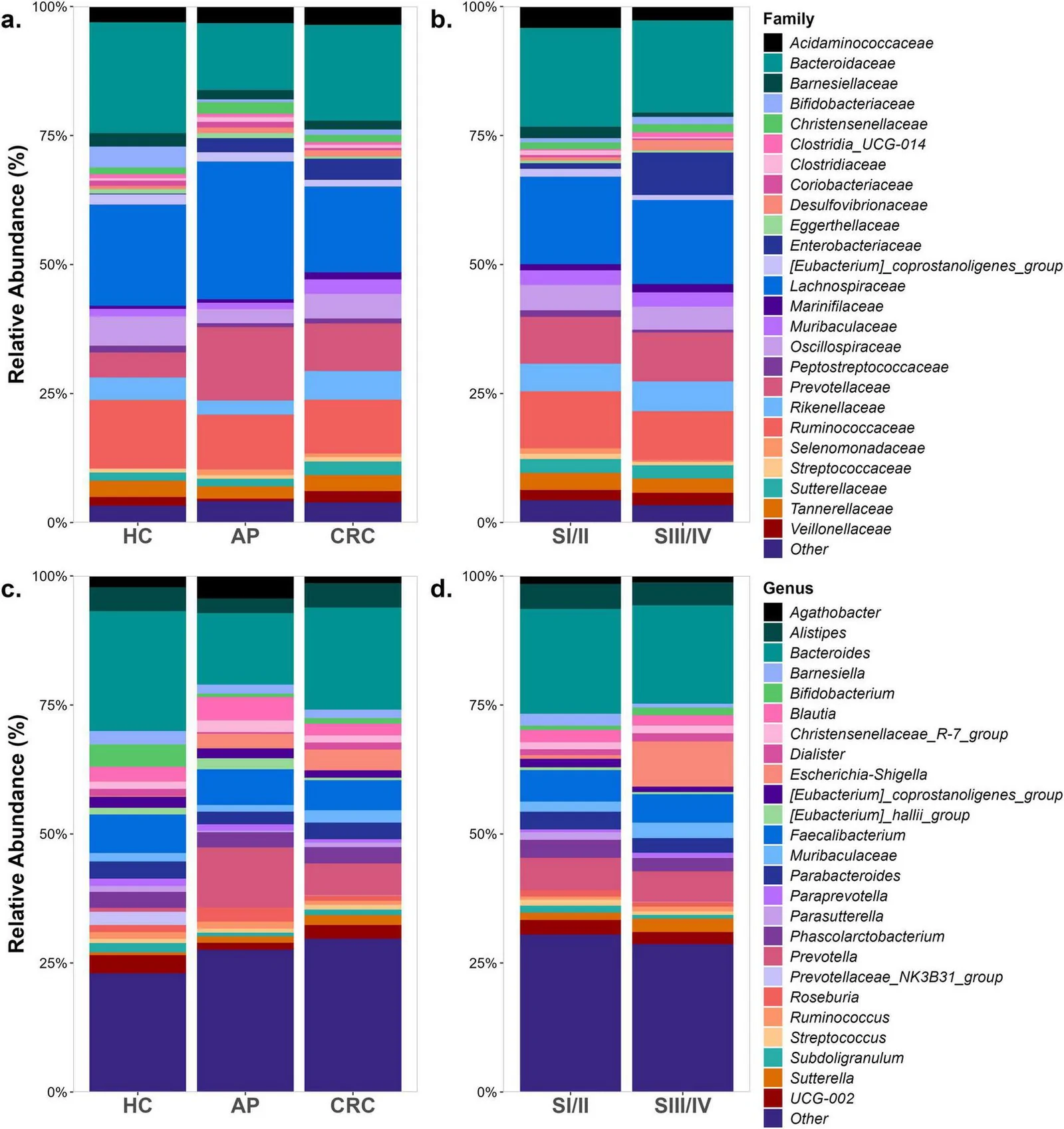
Community structure of stool microbiota communities in different clinical groups and cancer staging of the Portuguese cohort. Top panel shows the relative abundance at the family level, highlighting the 25 most abundant families (remaining families shown as “Other”): **(a)** per clinical group, comparing HC, AP, and CRC groups; and **(b)** along CRC staging, comparing SI/II and SIII/IV CRC stages. The bottom panel depicts the average relative abundance of the 25 most abundant genera (remaining genera shown as “Other”): **(c)** in each clinical group, comparing HC, AP, and CRC groups; and **(d)** along CRC staging, comparing SI/II and SIII/IV stages of CRC. HC, healthy controls; AP, Adenoma patients; CRC, colorectal cancer; SI/II and SIII/IV are, respectively, the CRC stages I/II and III/IV.

The stool microbiota in all groups was dominated by the Firmicutes and Bacteroidota phyla that jointly accounted for > 90% of relative abundance, whereas Proteobacteria, Actinobacteriota, and Desulfobacterota were less represented (Figure 2a). A few subtle shifts were however observed along the healthy-adenoma-carcinoma sequence. Actinobacteriota tended to decrease from HC to CRC, whereas Proteobacteria showed a notorious increase, reaching its highest relative abundance in advanced-stage CRC (SIII/IV) (Figure 2b). Desulfobacterota was relatively stable in most groups, but increased over threefold in CRC SIII/IV (∼2%) (Figure 2b). At the family level, Bacteroidaceae and Lachnospiraceae were the most abundant taxa in all groups, while Prevotellaceae was more dominant in AP (Figures 3a,b). Bifidobacteriaceae was substantially depleted in AP (0.56%) and CRC (∼1%) compared to HC (∼4%), and Ruminococcaceae tended to decrease along the HC-AP-CRC sequence. At the genus level, *Bacteroides* was predominant in all groups, and in HC it was followed by *Faecalibacterium* (∼6.9%) and *Alistipes* (∼4.3%) (Figures 3c,d). AP samples were more represented by *Blautia*, *Agathobacter*, and *Roseburia* genera. Notably, the relative abundance of *Bifidobacterium* and *Parasutterella* reduced in AP and CRC, reaching the lowest relative abundance at SIII/IV (0.11%). *Prevotella* relative abundance surged in disease groups, peaking in AP (∼10.9%); and *Escherichia-Shigella* and *Sutterella* showed a progressive proliferation along the adenoma-carcinoma sequence, being particularly high in CRC SIII/IV.

**FIGURE 4:**
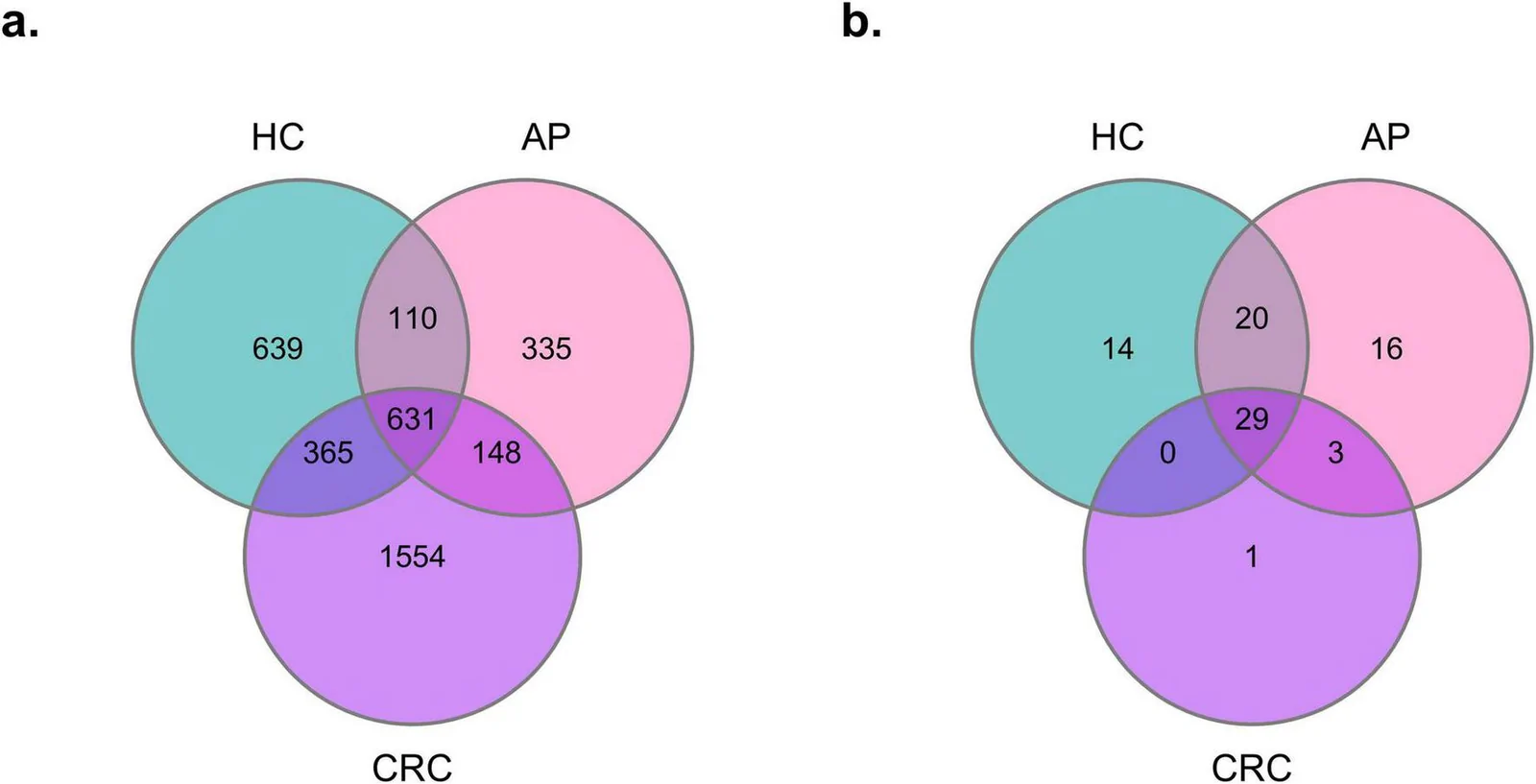
Total sequenced ASVs and core microbiota of all clinical groups of the Portuguese cohort. **(a)** Venn diagram of shared ASVs. **(b)** Venn diagram of shared and unique core ASVs (prevalence > 75%). HC, healthy controls; AP, adenoma patients; CRC, colorectal cancer patients.

In order to complement the characterization of the stool microbiota community structure across the clinical groups, we have assessed the distribution of shared and core microbiota patterns, following Venn and core microbiota analyses, respectively. Venn analysis revealed that most HC- and AP-associated ASVs were shared between at least two groups, while a large number was uniquely detected in the CRC group (1,554 ASVs) (Figure 4a). A total of 631 ASVs were common to all groups, being consistent with the presence of a conserved microbial community. The core microbiota (i.e., ASVs present in > 75% of subjects per group) was represented by 63, 68, and 33 core ASVs in HC, AP and CRC, respectively (Figure 4b). Twenty-nine ASVs were shared among all groups (Supplementary Table S3), while CRC exhibited only one unique core ASV that was assigned to the *Flavonifractor* genus.

#### 3.2.4 Differentially abundant microbial features associated with CRC

**FIGURE 5:**
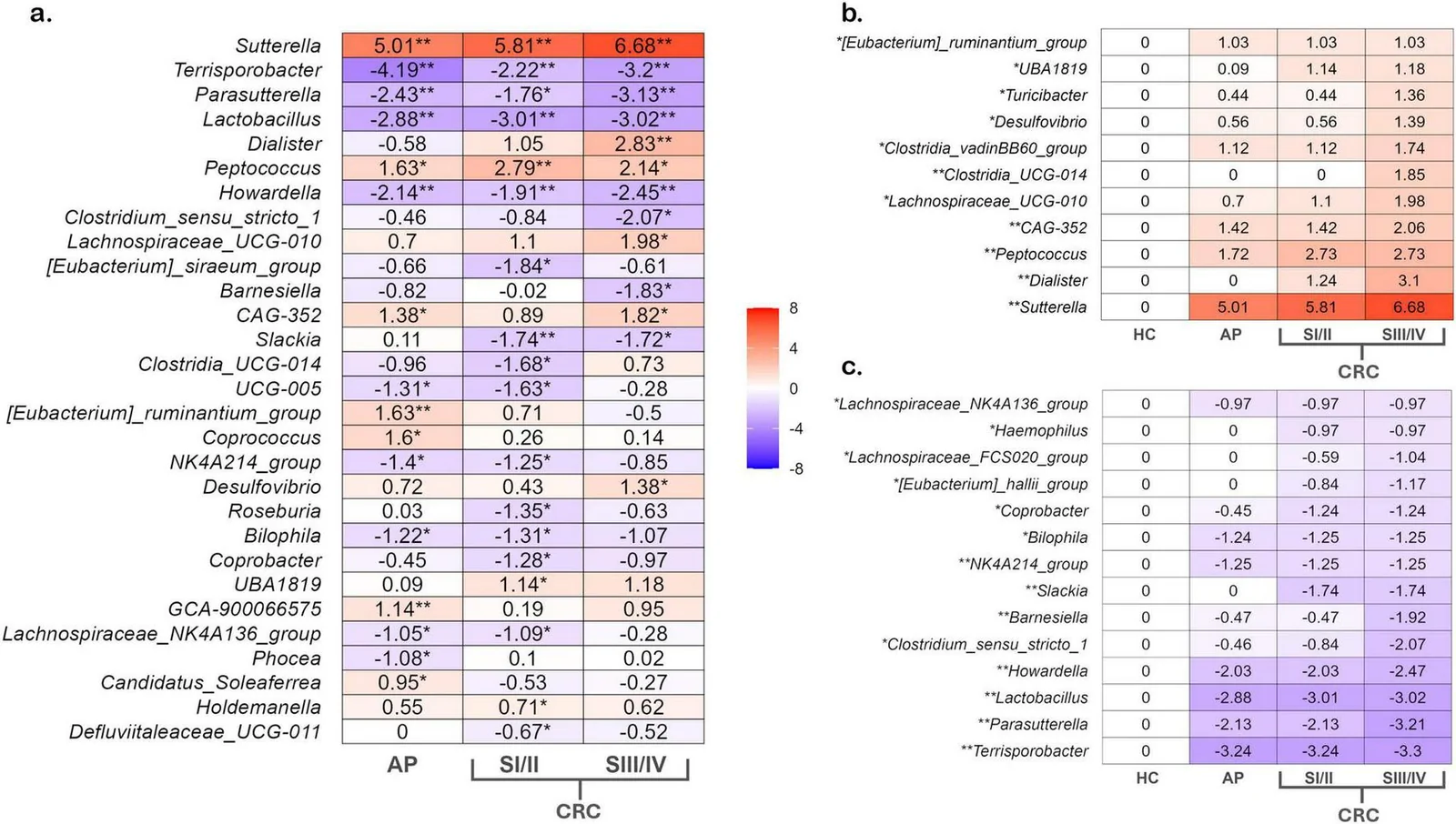
Results of ANCOM-BC2 obtained for the Portuguese cohort dataset. **(a)** Global analysis, evaluating differential abundance of bacterial genera. Right panels show results of pattern analysis, evaluating significant trends of **(b)** increasing and **(c)** decreasing abundance of bacterial genera throughout CRC staging. The numbers represent log fold-change values (LFC, natural log) of taxa abundance in AP and CRC stages (SI/II and SIII/IV) comparatively to HC (reference group). Cells are color-coded on a gradient, where red indicates increased abundance and purple/blue indicates decreased abundance. LFC values followed by an asterisk (*) represent significant changes without adjustment for multiple testing correction (*p* < 0.05), whereas values followed by two asterisks (**) are statistically significant after adjustment for mixed directional false discovery rate (Benjamini-Hochberg method, *q* < 0.05). HC, healthy controls; AP, adenoma patients; CRC (colorectal cancer) stages I/II, SI/II; and III/IV, SIII/IV.

DA analysis using ANCOM-BC2 adjusted for covariates with a 10%-prevalence filter detected multiple taxa significantly altered in AP and CRC comparatively to HC (Figure 5a, *p* < 0.05). In the global analysis, *Peptococcus* and *Suterella* were significantly enriched in all disease groups, and the latter genus assumed an effect size increase from AP (LFC = 5.01) up to CRC SIII/IV (LFC: 5.81–6.68), sustaining a potentially reliable association of *Sutterella* with CRC, even after false positive control (Figure 5a). On the other hand, *Terrisporobacter* (LFC: -2.22 to -4.19), *Parasutterella* (LFC: -1.76 to -3.16), *Lactobacillus* (LFC: -2.88 to -3.02), and *Howardella* (LFC: -1.91 to -2.45) were consistently depleted in AP and CRC staging groups, although *Parasutterella* lost significance in CRC SI/II after FDR correction (*q* > 0.05).

These associations were reinforced by ANCOM-BC2 trend analysis. *Suterella*, *Peptococcus*, and CAG-352 were monotonically increased in all disease groups after multiple testing correction (Figure 5b), while *Terrisporobacter*, *Parasutterella*, *Lactobacillus*, *Howardella*, *Barnesiella*, and NK4A214_group displayed a monotonic decrease along the adenoma-carcinoma sequence (Figure 5c). Additional stage-specific variations were also depicted. For instance, *Dialister* and *Clostridia*_UCG-014 were differentially enriched in CRC, particularly in SIII/IV, but not in AP (*Dialister*) or CRC SI/II (*Clostridia*_UCG-014) (LFC = 0; *q* > 0.05).

Pairwise ANCOM-BC2 provided a clearer resolution of these associations, confirming the enrichment of *Terrisporobacter* in CRC SI/II (LFC = 1.97) and *Dialister* in CRC SIII/IV (LFC = 3.41), as well as depletion of *Slackia* in both CRC stages (LFC ∼-1.8), all relatively to AP (Supplementary Figure S4).

Although microbial community variations among clinical groups was not significant (as per beta diversity), covariate-adjusted ANCOM-BC2 output a few taxa seemingly associated with CRC progression.

### 3.3 Assessment of the microbial features’ biological relevance

In order to further assess the feasibility and robustness of the disease-associated microbial features detected, we have performed cross-method and sensitivity analyses comparing: (i) adjusted versus unadjusted DA method (ANCOM-BC2) for covariates; (ii) different DA models; (iii) non-filtered versus 10%-filtered datasets for taxa prevalence; (iv) multiple testing correction for false positives; (v) matched subsets to reduce the effect size of potential confounding variables (age, sex and BMI) when applying DA models. In a broad view, there was a considerable agreement between DA frameworks regarding key disease-associated microbial taxa, although the amount, magnitude and statistical significance of the associations varied according to the DA and statistical method, prevalence filtering condition, and cohort matching analyzed.

In a first approach, we have compared the outcome of covariate-adjusted (Figure 5a) versus unadjusted (Supplementary Figure S5) ANCOM-BC2 (10%-prevalence filtered) to evaluate the influence of confounders in DA profiles. In the unadjusted model, only *Prevotella* increase (LFC = 3.48; *q* = 0.042) and *Terrisporobacter* decrease (LFC = -2.23; *q* = 0.042) in AP were significantly depicted after FDR correction (Supplementary Figure S5). Of these, solely *Terrisporobacter* was detected by the adjusted model, assuming a significant negative trend in all disease groups (Figure 5c). Other taxa were also commonly identified by both models, being either differentially enriched (e.g., *Sutterella*, *Desulfobrio*, GCA-900066575) or depleted (e.g., *Barnesiella*, *Parasutterella*) in AP or late CRC stage; however, they generally lost significance after multiple testing correction in the unadjusted model (Supplementary Figure S5).

**FIGURE 6:**
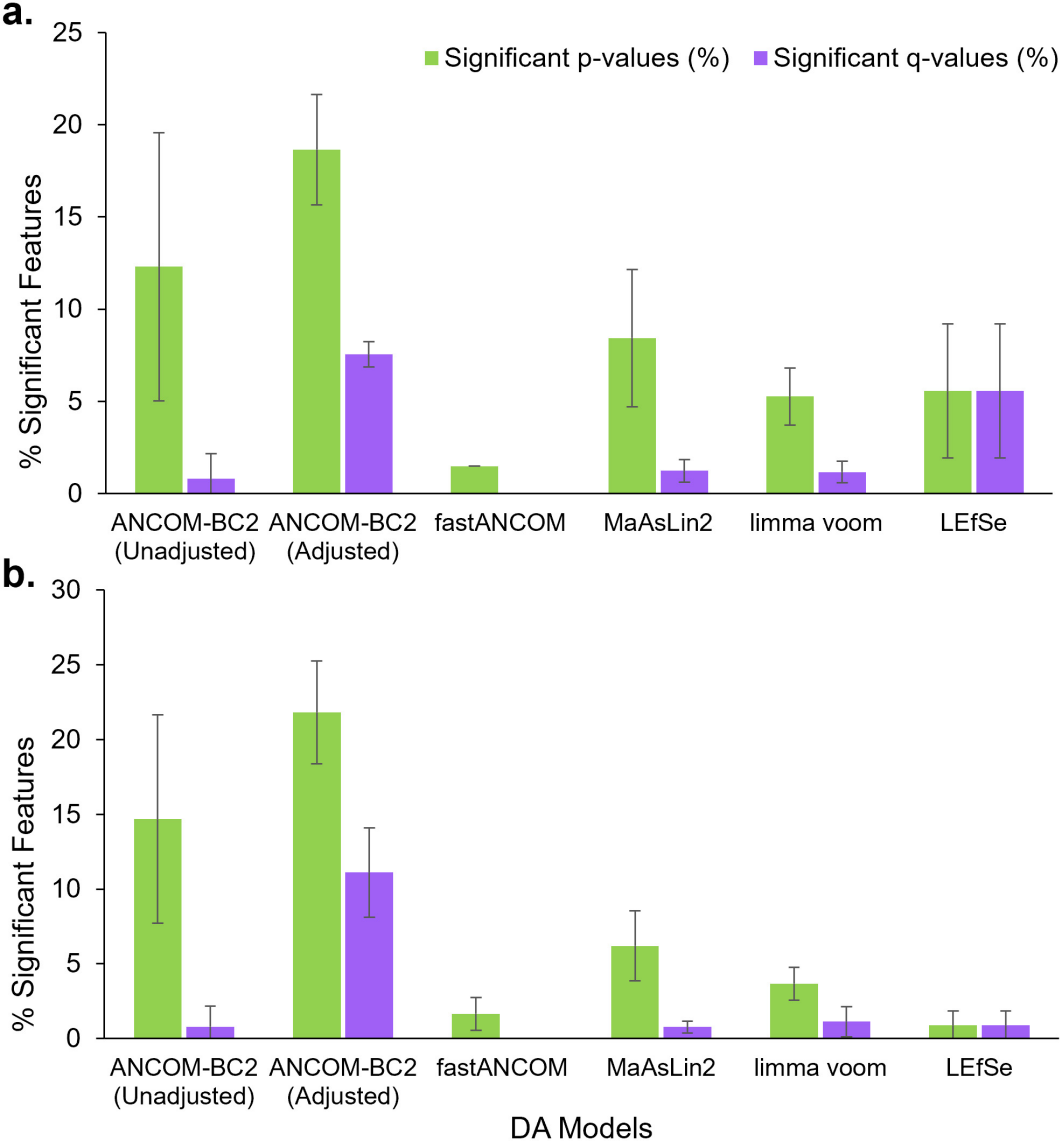
Significant features identified in **(a)** data filtered by prevalence (10%) and **(b)** unfiltered data, per differential abundance (DA) methodology applied to the Portuguese cohort dataset: ANCOM-BC2 (unadjusted), ANCOM-BC2 (adjusted for covariates), fastANCOM, MaAsLin2, limma voom, and LEfSe. Bars represent mean percentage of significant *p*-values in all groups (*p* < 0.05) and mean percentage of significant *q* values (*q* < 0.05 in all methods, except MaAsLin2, where significance threshold was *q* < 0.25) ± standard deviation.

We have then examined how distinct DA methods affected the amount and profiles of microbial taxa significantly detected. ANCOM-BC2 retrieved the highest percentage of significant taxa, both in unfiltered (14.68 ± 6.98% to 21.83 ± 3.44%) or 10%-prevalence filtered analyses (12.30 ± 7.27% to 18.65 ± 3.00%) (Figure 6 and Supplementary Table S4). After multiple testing correction, the adjusted ANCOM-BC2 also presented the highest statistical power, retrieving ∼11% of significant taxa, whilst fastANCOM (0%), MaAsLin2 (0.78 ± 0.39% to 1.23 ± 0.62%), and LEfSe (0.89 ± 0.96%) detected the lowest amount (Supplementary Table S4). In what concerns the microbial features identified, in the global analyses, *Sutterella* surged as the most reproducible CRC-associated taxon, showing a progressive and significant increase after FDR correction, from AP to advanced CRC by all DA models (Figure 5a and Supplementary Figures S6, S7) except fastANCOM (not significant after FDR correction; Supplementary Figure S8) and LEfSe (absent taxa; Supplementary Figure S9). Enhancement of *Desulfovibrio* abundance was also significant, particularly in late-stage CRC, as per ANCOM-BC2, fastANCOM, limma voom, and LEfSe methods (Figure 5a and Supplementary Figures S7–S9); whilst *Peptococcus* increase was significantly detected only by ANCOM-BC2 and limma voom. However, upon multiple testing correction, *Desulfovibrio* was found significant only in CRC SIII/IV by limma voom (Supplementary Figure S7); and *Peptococcus* was kept significant just in CRC SI/II by ANCOM-BC2 (Figure 5a). LEfSe supported *Desulfovibrio* enrichment in CRC, and also detected the enhancement of *Prevotella*, *Agathobacter*, and *[Eubacterium]_hallii*_group in AP, and *Fusobacterium* in CRC (Supplementary Figure S9). Depleted taxa, however, showed greater variability across DA outputs. *Terrisporobacter*, *Parasutterella*, *Lactobacillus*, *Howardella*, and *Slackia* were recurrently decreased in disease groups (particularly in AP and SI/II CRC) according to ANCOM-BC2 (Figures 5a,c). In turn, fastANCOM, limma voom, and MaAsLin2 (Supplementary Figures S6–S8), had more congruently detected *Adlercreutzia* and *Parasutterella* decrease in adenoma and/or late stages of CRC; though these two latter associations were just reinforced by MaAsLin2, after FDR correction (Supplementary Figure S6a).

Prevalence filtering had a low impact on overall DA taxa profiles. MaAsLin2 output was identical for non-filtered and filtered datasets (Supplementary Figures S6a,c). The relevant disease-associations determined for *Sutterella*, *Desulfovibrio*, *Dialister*, *Parasutterella*, and *Adlercreutzia* were steadily highlighted in ANCOM-BC2, fastANCOM, MaAsLin2, or limma voom outputs after filtering (Figures 5a,b and Supplementary Figures S6a, S7, S8, respectively), suggesting that the identification of these microbial features was not constrained by eventual dataset sparsity in the unfiltered analyses (Supplementary Figures S6c, S9, S10–S12). LEfSe consistently associated *Desulfovibrio* with SIII/IV CRC in filtered and unfiltered datasets; but the filtered analysis yielded two additional significant taxa in SI/II, *Parasutterella* and *[Ruminococcus]_torques*_group (Supplementary Figure S9). Nevertheless, LEfSe does not conduct false positive rate discovery, meaning that its results grant limited interpretability.

We next investigated the impact of age, sex, and BMI confounding variables on the microbe-disease associations retrieved by the DA models applied to matched sub-cohorts. LEfSe was excluded from these analyses due to its inability to adjust for covariates. Age-matched analyses reduced enormously the number of significant taxa detected by ANCOM-BC2, fastANCOM, and MaAsLin2, whilst sex- and BMI-matched subgroups generally called a larger amount of disease-associated taxa (Supplementary Figures S6–S8, S10–S13). The small size of age-matched subgroup might have biased and reduced the statistical power of the analyses. Still, such discrepancies were not observed for limma voom (Supplementary Figures S7, S12), which normally outputs high false positive rates due to the limited performance of TMM normalization in sparse datasets (Robinson and Oshlack, 2010). Throughout the matched analyses, *Sutterella* was weakened (by fastANCOM), lost (by ANCOM-BC2 and MaAsLin2) or enriched after FDR correction (by filtered limma voom). Therefore, despite the reproducibility of this genus it may be partially sensitive to cohort demographics and/or to a lower statistical power after cohort stratification. *Desulfovibrio* and *Dialister* were also significantly increased in sex- and BMI-matched groups by ANCOM-BC2 and limma voom models (Supplementary Figures S7, S13). *Parasutterella* presented a stably depleted pattern by most DA tools (ANCOM-BC2, fastANCOM, limma voom, and MaAsLin2), as well as across matched subsets, particularly by ANCOM-BC2 (for sex-matched) and MaAsLin2 after false positive controlling (Supplementary Figures S6–S8, S10–S13). This suggests that *Parasutterella* is seemingly less sensitive to confounders, and may be a comparatively robust CRC-associated feature within the Portuguese cohort.

**FIGURE 7:**
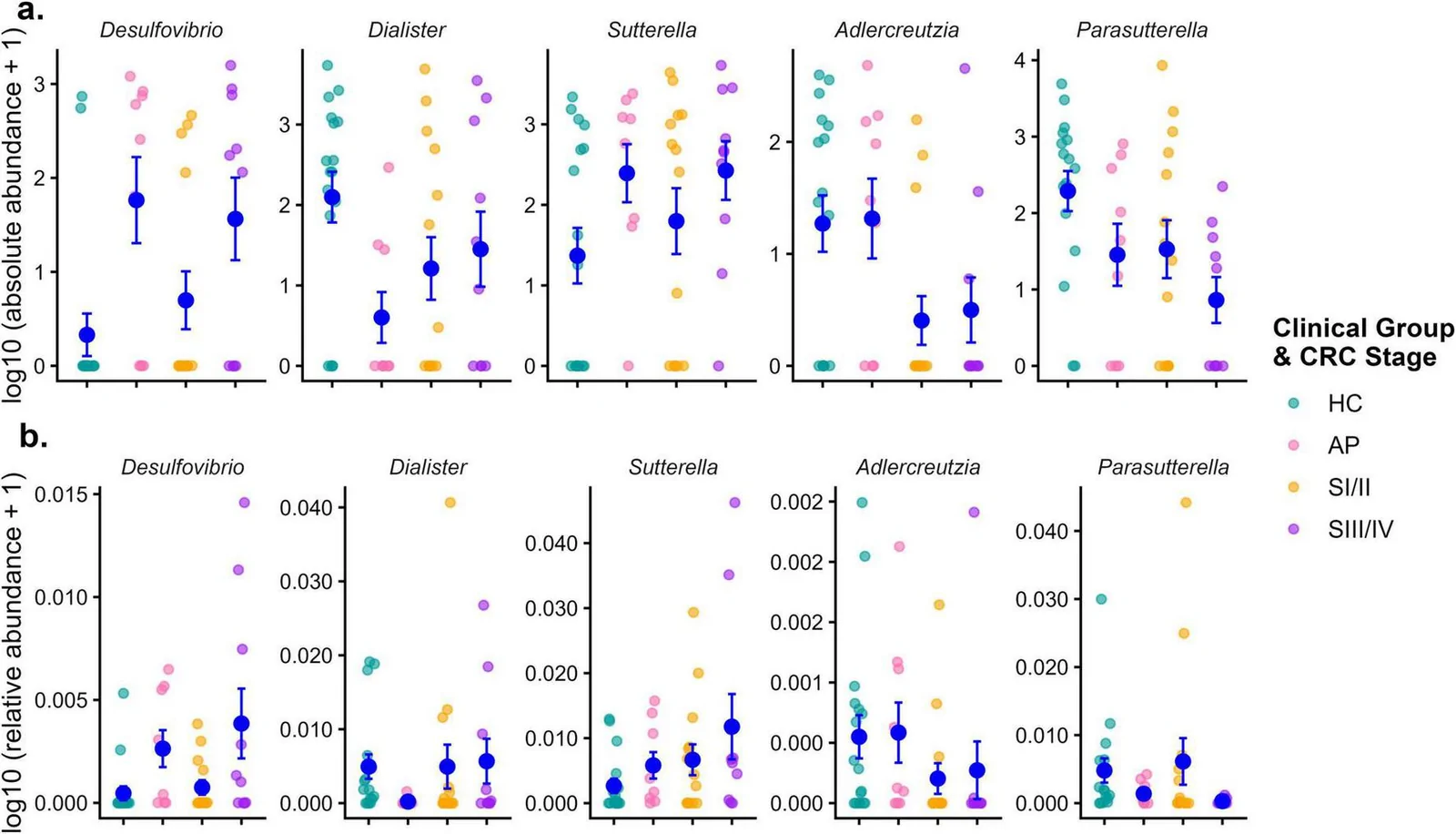
Graphical representation of **(a)** log absolute abundance and **(b)** log relative abundance of significantly increased (*Desulfovibrio, Dialister, Sutterella*) and significantly decreased (*Adlercreutzia*, *Parasutterella*) genera, as per differential abundance analysis, plotted by clinical group & CRC stage of the Portuguese cohort. HC, healthy controls; AP, adenoma patients; CRC (colorectal cancer) stages I/II: SI/II; and III/IV: SIII/IV.

Figure 7 displays the logarithmized absolute and relative abundances of some taxa that were identified across the DA analyses performed. Coherently, *Sutterella* and *Desulfovibrio* were increased from HC to CRC, as well as *Parasutterella* and *Adlercreutzia* were consistently and more clearly decreased along the AP-CRC sequence. These trends are identical whether absolute or relative abundances are used, supporting the reproducibility of these features as potential biomarker candidates of CRC progression.

### 3.4 Microbial features’ robustness among distinct datasets

#### 3.4.1 Microbial community structure across countries

To assess the reproducibility of the microbial features identified in the Portuguese cohort, an exploratory cross-cohort analysis was undertaken against four publicly available stool 16S rRNA gene sequencing datasets from France, Ireland, Italy, and United States/Canada (Table 3). Within individual cohorts, alpha-diversity metrics did not differ significantly between clinical groups (Supplementary Table S5).

On the contrary, beta diversity analyses performed on the cohort-pooled dataset revealed significant differences in microbiome structure across datasets (PERMANOVA, *p* = 0.001, Supplementary Figure S14). However, no significant clustering was observed by country, evidencing a considerable overlap between datasets, both when HC and diseased (AP and CRC) groups were analyzed separately (Supplementary Figures S14a–c; *p* = 0.001). Hence, although the country of origin or cohort significantly contributed to microbiome variation, the observed differences were not sufficiently divergent to support the existence of clear population-specific microbial profiles.

Univariate PERMANOVA detected significant individual effects of the country of origin, clinical group, and age group on the microbiome structure (Supplementary Table S5). In the multivariate model, however, the age group lost significance after adjustment. The country of origin explained the largest proportion of variation in community composition (*R*^2^: 20.59–23.25%), while the clinical group showed a smaller, but still statistically significant association (*R*^2^: 0.61–0.96%, *p* = 0.025, *q* = 0.05). Age group and sex were not independently associated with beta diversity in the multivariate model. Beta dispersity analyses reinforced the significant heterogeneity within the clinical group (*p* = 0.011), country of origin, and age group (*p* = 0.001). Such heterogeneity may likely reflect the combined influence of biological and methodological variability among studies, namely differences in sample processing and storage, 16S rRNA gene target regions, cohort size, and unequal representation of clinical groups. Thereby, the multi-cohort analyses should be interpreted as exploratory evaluations of cross-dataset reproducibility, rather than definitive evidence of distinct geographic microbial signatures.

#### 3.4.2 Shared and cohort-specific CRC signatures

**FIGURE 8:**
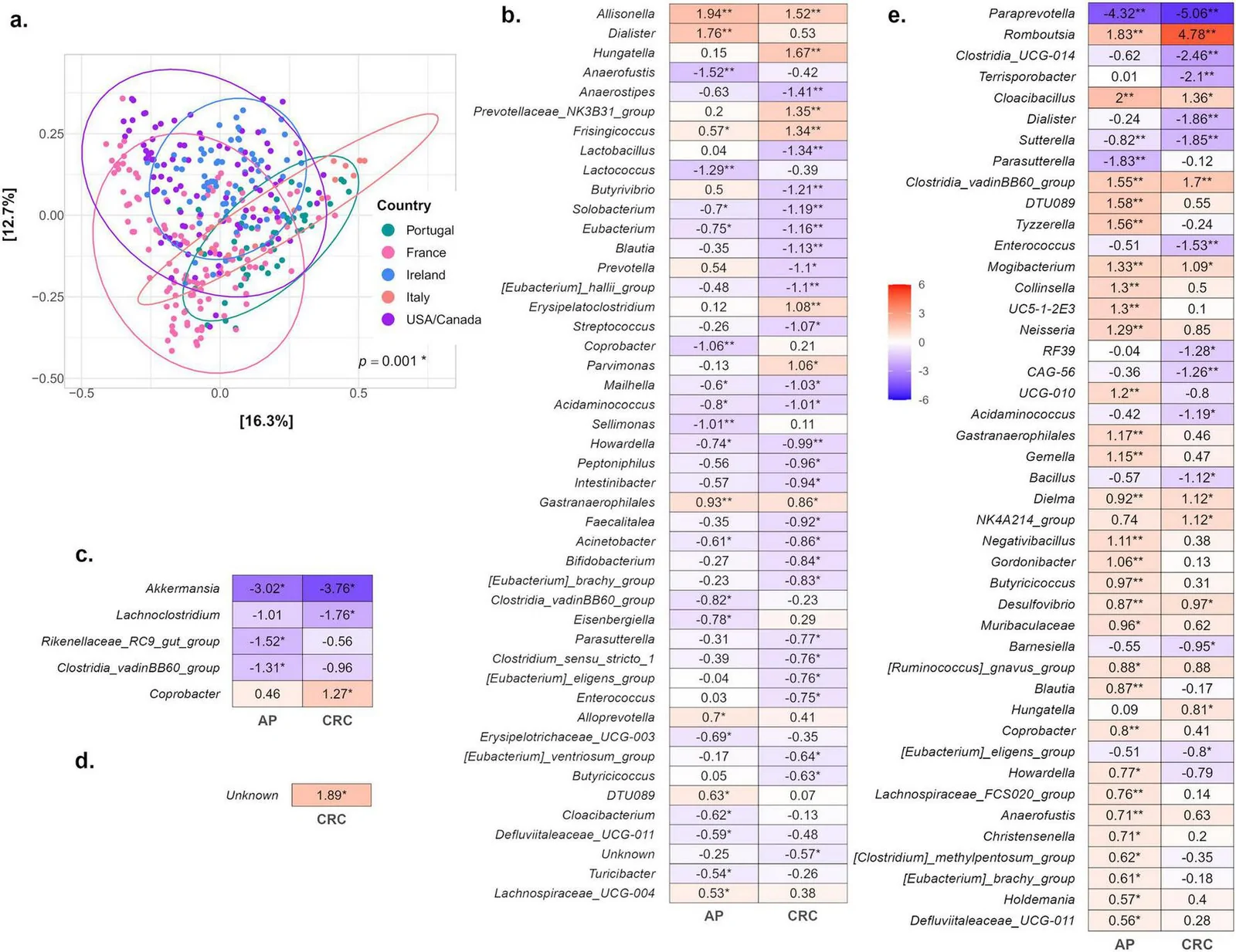
Results of analysis of external cohorts. **(a)** PCoA ordination of beta diversity analysis through Bray-Curtis dissimilarity, plotted by country of origin/cohort. Healthy (Supplementary Figure S7c) and diseased (AP and CRC patients, cf. Supplementary Figure S7d) subjects were also plotted by country. ANCOM-BC2 analysis performed for each cohort: **(b)** France; **(c)** Ireland; **(d)** Italy; **(e)** United States/Canada. The numbers represent log fold-change values (LFC, natural log) of taxa abundance in AP and CRC comparatively to HC (reference group). Cells are color-coded on a gradient, where red indicates increased abundance and purple/blue indicates decreased abundance. LFC values followed by an asterisk (*) represent significant changes without adjustment for multiple testing correction (*p* < 0.05), whereas values followed by two asterisks (**) are statistically significant after adjustment for mixed directional false discovery rate (Benjamini-Hochberg method, *q* < 0.05). HC, healthy controls; AP, adenoma patients; CRC, colorectal cancer patients.

ANCOM-BC2 framework was selected for conducting DA analysis within each cohort independently, due to its suitability for multigroup compositional analyses with covariate adjustment (Figures 5a, 8b–e). ANCOM-BC2 detected a few microbial features with comparable AP- or CRC-associated trends between cohorts. After multiple testing correction, *Dialister* was significantly enriched in the Portuguese (in CRC) and French (in AP) cohorts, while *Lactobacillus* and *Howardella* were significantly depleted in all disease groups analyzed from Portugal, and in the CRC group from France (Figures 5, 8b–e, respectively). Portugal and United States/Canada shared microbial taxa with decreasing abundances for *Parasutterella* in AP and *Terrisporobacter* in CRC. *Dialister*, *Sutterella*, and *Peptococcus* were also common to the latter two datasets, though they displayed opposite directional trends (Figures 5, 8b–e, respectively), hence highlighting the context dependency of several microbial associations. No significant taxa were shared between cohorts from Portugal, Italy, and Ireland. These findings indicate that while a limited number of AP- and/or CRC-associated taxa were reproducible across independent datasets, most microbial features appeared to be cohort-specific, possibly influenced by the demographic and methodological heterogeneity between studies.

## 4 Discussion

Increasing evidence linking gut microbiota dysbiosis, intestinal inflammation, and colorectal carcinogenesis (White and Sears, 2024), has intensified the search for microbial signatures associated with colorectal adenoma and carcinoma. However, the substantial inter-individual variability, cohort heterogeneity, and methodological differences among studies, introduce biases in the identification of feasible and universal microbial markers of CRC progression. As a result, spurious associations may be deduced, which might not exactly reflect real changes in microbiome composition related with malignant transformations and tumor development (Nearing et al., 2021). In this context, the present study characterized stool microbiota alterations along the adenoma-carcinoma sequence in a Portuguese cohort, and evaluated the robustness and reproducibility of diseases-associated microbial features across different constraining factors or conditions.

The gut microbiome diversity of the Portuguese cohort was partially coherent with the profiles described in the literature for microbial community changes related with adenoma and CRC. We confirmed that colorectal carcinogenesis is not necessarily followed by steep changes in the overall community richness and diversity (non-significant alpha diversity; Figure 1a and Supplementary Table S1), but rather by selective ecological restructuring of specific microbial populations, as previously observed (Thomas et al., 2019; Yachida et al., 2019; Piccinno et al., 2025). As per the taxonomic profiling, there was a predominance of Bacteroidota and Firmicutes in all clinical groups (Figure 2), in accordance with other studies on CRC microbiomes (e.g., Gao et al., 2020; Sun et al., 2020; Chen et al., 2021; Wu et al., 2021). The progressive enrichment of Proteobacteria, particularly in advanced CRC, may corroborate the association of this phylum with gut inflammation, dysbiosis (Hiippala et al., 2016), and CRC progression (Gao et al., 2020; Sun et al., 2020). Conversely, the reduction of Actinobacteriota, especially Bifidobacteriaceae (Figures 3a,b), together with the decrease of relative abundance of known short-chain-fatty-acid-producing genera (e.g., *Bifidobacterium*, *Faecalibacterium*, *Subdoligranulum*), suggests a gradual disruption of beneficial microbial populations linked to gut health and homeostasis (Kumar et al., 2023; Ruiz-Saavedra et al., 2023; Singh et al., 2023; Figure 3c). Increased abundance of *Prevotella* in AP samples may additionally reflect its role in early inflammatory and dysbiostic changes associated with neoplastic transition (Precup and Vodnar, 2019). Accordingly, core microbiota analysis suggested that CRC-driven dysbiosis is essentially characterized by discrete changes in specific microbial taxa than by the emergence of a conserved CRC-associated microbiome (Figure 4; Falony et al., 2016).

In agreement with this observation, covariate-adjusted ANCOM-BC2 identified a few microbial features exhibiting alterations along disease status. While some taxa displayed stage-specific patterns (e.g., *Dialister* and *Slackia*; Figures 5a,b); the strongest effect sizes and most consistent associations were generally observed for taxa monotonically increasing (e.g., *Sutterella*) or decreasing (e.g., *Parasutterella*, *Terrisporobacter*, *Lactobacillus*, *Howardella*) (Figure 5c) from HC to adenoma, early- and/or advanced-CRC stages.

Although beta diversity analyses revealed significant changes in the microbial community structure along AP-CRC sequence, these effects were attenuated after covariate adjustment (Figure 1b and Supplementary Table S1), indicating that microbiome composition variation was in part triggered by demographic heterogeneity and other confounding variables, as largely discussed by other authors (e.g., Thomas et al., 2019; Vieira-Silva et al., 2020; Liu et al., 2023; Tito et al., 2024). This highlights the importance of controlling the co-action of host, demographic and methodological confounders when inferring microbial signatures of disease status (Wirbel et al., 2019; Nearing et al., 2021).

Given the recognized influence of methodological aspects on the microbiome DA analysis (Nearing et al., 2022; Wirbel et al., 2024; Karwowska et al., 2025), we have next systematically evaluated the robustness of the identified microbial features across different constraining factors and testing conditions.

The comparison of unadjusted (Supplementary Figure S5) versus covariate-adjusted (Figure 5a) ANCOM-BC2 with a 10%-prevalence filter, retrieved a partial overlap of disease-associated taxa between the two model configurations, represented by the enrichment of *Sutterella*, *Prevotella*, and *Desulfovibrio*, and the depletion of *Terrisporobacter*, *Parasutterella*, and *Barnesiella*. But, when correcting for false discoveries, only *Terrisporobacter* was detected by both models, being significantly depleted either in adenoma (unadjusted model) or throughout the adenoma-carcinoma sequence (adjusted model). Overall, the covariate-adjusted ANCOM-BC2 retained a higher proportion of significant taxa after FDR control (Figure 5a), supporting previous observations that compositional-aware models adjusted for confounding variables improve the reliability and interpretability of DA analyses (Lin and Peddada, 2024; Wirbel et al., 2024).

Recent benchmarking studies demonstrated that DA models frequently retrieve distinct results, due to their intrinsic compositional assumptions, sparsity handling methods, normalization and transformation procedures, and multiple testing strategy (e.g., Nearing et al., 2022; Wirbel et al., 2024). Accordingly, we obtained somewhat different outputs by the DA tools employed, although several taxa were commonly identified as significant by the majority of the DA models, depicting a similar directional effect across the testing conditions assessed. A clear example of this was *Sutterella*, which was the most reproducible AP- and CRC-associated feature, being significantly enriched by most DA tools (not by LEfSe) and testing conditions, including with prevalence filtering (except for fastANCOM) and FDR correction. *Peptococcus* and *Desulfovibrio* were as well recurrently and significantly associated with CRC by two (ANCOM-BC2, limma voom) and three (ANCOM-BC2, fastANCOM, limma voom) models, respectively (Figure 5 and Supplementary Figures S7, S8). However, upon false positive control, *Peptococcus* remained significantly enriched only in early disease stages (AP, SI/II) by ANCOM-BC2 (Figure 5), and *Desulfovibrio* was more significantly associated with late CRC as per limma voom. Among the depleted taxa, *Parasutterella* (ANCOM-BC2, filtered fastANCOM, limma voom; Figure 5 and Supplementary Figures S7, S8) and *Adlercreutzia* (MaAsLin2, limma voom, unfiltered fastANCOM; Supplementary Figures S6, S7, S11) were more commonly called across most unfiltered and/or filtered DA tools.

Interestingly, in age-, sex-, and/or BMI-stratified cohorts, *Sutterella*, *Parasutterella* (absent in age subset), or *Adlercreutzia* (only present in BMI subset) were still significantly detected by at least one DA method, suggesting their relative robustness against demographic confounders (Supplementary Figures S6–S8, S10–S13). Lower levels of *Parasutterella* were previously observed in CRC (Wang et al., 2012), and Clos-Garcia et al. (2020) pointed out *Adlercreutzia* as a potential early warning marker of CRC given its enrichment in AP, though being a contradictory directional effect as per the depletion observed in our study. *Dialister*, which was not a feature consistently detected across DA methods in the global dataset (Figure 5), emerged after cohort stratification in all matched subsets analyzed, by at least one DA model upon FDR correction (Supplementary Figures S6–S8, S10–S13), supporting previous studies linking this genus with dysbiosis in CRC patients (Regueira-Iglesias et al., 2023).

Although usually regarded as a comparatively conservative compositional framework (Nearing et al., 2022), ANCOM-BC2 retrieved the highest percentage of significant taxa in the Portuguese cohort, followed by MaAsLin2, fastANCOM, limma voom, and LEfSe, either in unfiltered and filtered datasets for prevalence (Figure 6 and Supplementary Table S4). This outcome may however reflect the suitability of ANCOM-BC2 for multigroup microbiome analyses with covariate adjustment and mixed directional FDR control for multiple pairwise comparisons, particularly in datasets characterized by pattern-associated abundance variations over ordered categories (e.g., disease stages), moderate effect sizes, and considerable sparsity, as observed in the Portuguese cohort (Lin and Peddada, 2024). In contrast, fastANCOM (log-transformed ratios), MaAsLin2 (CLR transformation), and limma voom (TMM transformation and data normalization by the upper-quartile) may exhibit reduced statistical power in relatively small and sparse microbiome datasets, being affected by data distribution, and normalization and transformation methods used (Ritchie et al., 2015; Hawinkel et al., 2019; Mallick et al., 2021; Zhou et al., 2022; Wirbel et al., 2024). LEfSe is even more critical since it does not adjust for covariates and filters features’ significance by effect size instead of using FDR correction, being thereby vulnerable to false positive associations (Nearing et al., 2022). This was particularly evident for *Fusobacterium*, which was not significantly linked to a CRC stage after FDR correction in any of the analyses, in agreement with recent discussions on the effectiveness and reliability of *Fusobacterium* as a CRC biomarker, as its derivation might have been biased by unaccounted confounders (Tito et al., 2024). Thus, LEfSe must be regarded as an exploratory tool instead of confirmatory, and given the inconsistencies in regard of the other DA tools, the interpretation of its results was severely downweighted.

In summary, *Sutterella* and *Desulfovibrio* were the most reproducible features associated with CRC across DA frameworks and matched subset analyses, both showing a progressive enrichment along the adenoma-carcinoma sequence. *Parasutterella* and *Adlercreutzia* were also consistently depleted features despite being more sensitive to covariates. Their absolute and relative abundance profiles followed directional trends (Figure 7), further sustaining their possible biological importance toward disease onset and CRC development associations. An additional finding with clinical relevance is that *Sutterella* and *Parasutterella* were differentially represented already in adenoma, and *Adlercreutzia* decrease occurred in early CRC stage (SI/II), supporting their potential as early-warning microbial markers of CRC malignant progression.

Considering the higher robustness of ANCOM-BC2 model throughout the DA and sensitivity analyses, covariate-adjusted, and prevalence-filtered testing performed, we have selected this tool to explore the reproducibility of our candidate microbial features across geographically-distinct datasets. The microbiome composition was strongly influenced by the country of origin (*p* = 0.001; Figure 8a and Supplementary Table S5) than by the clinical group (*p* = 0.003; Supplementary Table S5), in line with previous studies (Wirbel et al., 2019; Gülhan et al., 2024). Notwithstanding, the substantial overlap between datasets indicated that geographically different populations did not exhibit clearly segregated microbiome structures (Figure 8a and Supplementary Figure S14). Still, ANCOM-BC2 depicted a few reproducible microbial taxa among datasets (Figures 8b–e). After FDR correction, *Dialister* was commonly enriched in the Portuguese (in CRC) and French (in AP) datasets. In turn, *Parasutterella* and *Terrisporobacter* were coherently depleted in the Portuguese and United States/Canada datasets, both taxa showing more pronounced effect sizes linked to AP and CRC, respectively. Despite the substantial population- and study-related heterogeneity, some taxa, namely *Parasutterella*, may represent biologically relevant and reproducible signatures of adenoma precursor lesions and CRC development, strengthening their value for further validation steps in large-scale studies.

Despite the groundbreaking knowledge and outputs produced in this study, it has a few limitations, which were carefully taken into consideration. First, the Portuguese cohort size was relatively small, particularly for adenoma and early stage CRC groups, which might have constrained the statistical power for detecting subtle microbial associations. The uneven distribution of age and sex in the clinical groups reflected the epidemiological profile of CRC in Portugal, where the disease predominantly affects males and older individuals, also in agreement with usual CRC incidence trends (Elmadani et al., 2025). Although covariate-adjusted DA methods and matched sensitivity analyses were performed to mitigate the impact of demographic heterogeneities, confounding effects may not be fully excluded. In addition, potentially relevant variables, such as diet, smoking, alcohol use, and tumor location were not available and therefore could not be accounted for in the analyses. The use of 16S rRNA gene sequencing may restrict taxonomic resolution and functional inferences, though it is still deemed valid for taxonomic and diversity analyses of gut microbiome associated with CRC (Regueira-Iglesias, et al. 2023; Matchado et al., 2024). Moreover, the cross-sectional study precludes causal relationships between microbiome shifts and colorectal carcinogenesis. Nevertheless, cross-sectional microbiome studies are highly valuable for the exploratory identification of disease-associated microbial features exhibiting biologically meaningful effect sizes (Wirbel et al., 2019; Piccinno et al., 2025), which might then be targeted for subsequent validation (large-scale) studies.

In spite of these limitations, our study also presents strengths. The inclusion of clinically-distinct groups ranging from healthy individuals, to adenoma and CRC patients enabled the characterization of microbiota communities linked to the adenoma-carcinoma sequence. Additionally, we employed an approach that fostered a reliable contamination and covariate control, the use of multiple DA models, and performance of sensitivity analyses, which together had greatly strengthened the robustness assessment of the identified microbial features or signatures of adenoma and colorectal carcinoma. The exploratory integration of external datasets supported the limited reproducibility of selected microbial features across independent populations, evidencing a considerable inter-cohort heterogeneity.

In conclusion, this study provides the first characterization of stool microbiota alterations along the adenoma-carcinoma sequence in a Portuguese cohort, while assessing the robustness of disease-associated microbial signatures across multiple frameworks. Following a comprehensive statistical approach, we could successfully detect the gradual enrichment of *Sutterella* and *Desulfovibrio*, in parallel with the depletion of *Parasutterella* and *Adlercreutzia*, associated with adenoma and CRC progression. Furthermore, *Sutterella*, *Parasutterella* and *Adlercreutzia* were particularly altered in early disease stages, thereby sustaining their potential biologically-informative value for disease development monitoring. *Parasutterella* was also represented in some external datasets, but most microbial features were population-specific. Surprisingly, *Fusobacterium*, which has been broadly accepted as CRC biomarker, was not a significant microbial feature after covariate adjustment. Taking altogether, our study and achieved outcomes underlines the importance of integrating underrepresented populations, covariate-adjusted and compositional-aware methods, underscoring the need for standardized analytical strategies in microbiome studies focusing the derivation of feasible adenoma- and CRC-related microbial features. Future investigations should combine larger cohorts, longitudinal designs, shotgun metagenomics, and multi-omics approaches to further validate the translational and predictive power of the selected microbial features in colorectal cancer.

## Data Availability

The datasets generated for this study can be found in the Supplementary material and in the Zenodo repository (https://doi.org/10.5281/zenodo.17599189). Further inquiries can be directed to the corresponding author.

## Footnotes

1
https://mothur.org/MicrobiomeBiomarkerCRC/
