# Supplementary material for: Stool microbiota variations along the adenoma-colorectal carcinoma sequence — robustness of disease-associated microbial features
Source: Front Microbiol. 2026 Aug 20;17:1822941. doi: 10.3389/fmicb.2026.1822941 (PMC13539885; doi:10.3389/fmicb.2026.1822941)
Supplement: Supplementary file 1 [file Supplementary_file_1.pdf]

Supplementary Material

Changes in stool microbiota composition along the adenoma-colorectal carcinoma sequence and divergence of microbial features across cohorts

Ana Carvalho, Silvia Gschwendtner, Michael Schloter, Ana M. Gil, Catarina R. Marques

1 Supplementary Figures and Tables

1.1 Supplementary Figures

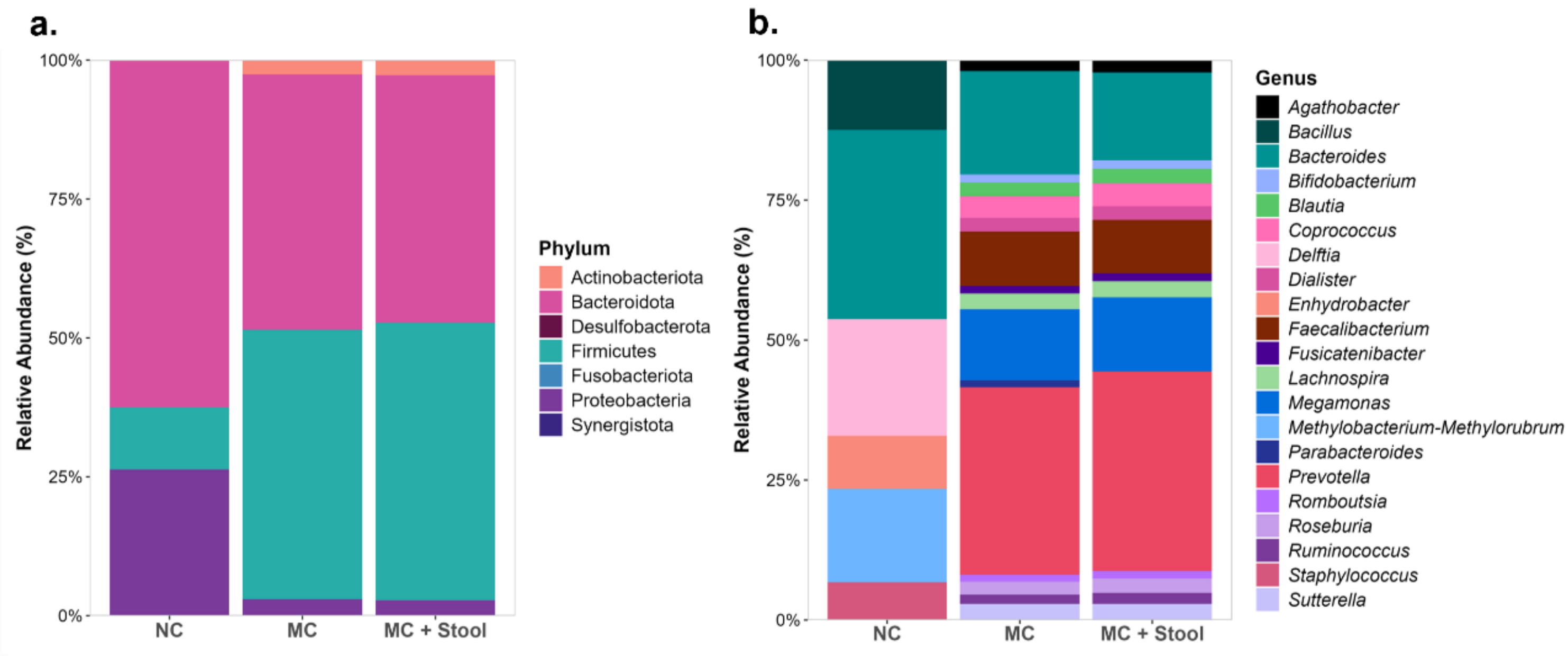

Figure S1. Bar plots represent relative abundance characterization of sequenced ASVs at the phylum **(a)** and genus **(b)** level of negative controls (NC), mock community sample (MC), and mock community mixed with a stool sample (MC + Stool) for validation of the sequencing methodology. A total of 58 reads were amplified from NC samples (*n* = 6); while 38,112 and 93,854 reads were amplified from MC (*n* = 1) and MC + Stool (*n* = 1), respectively.

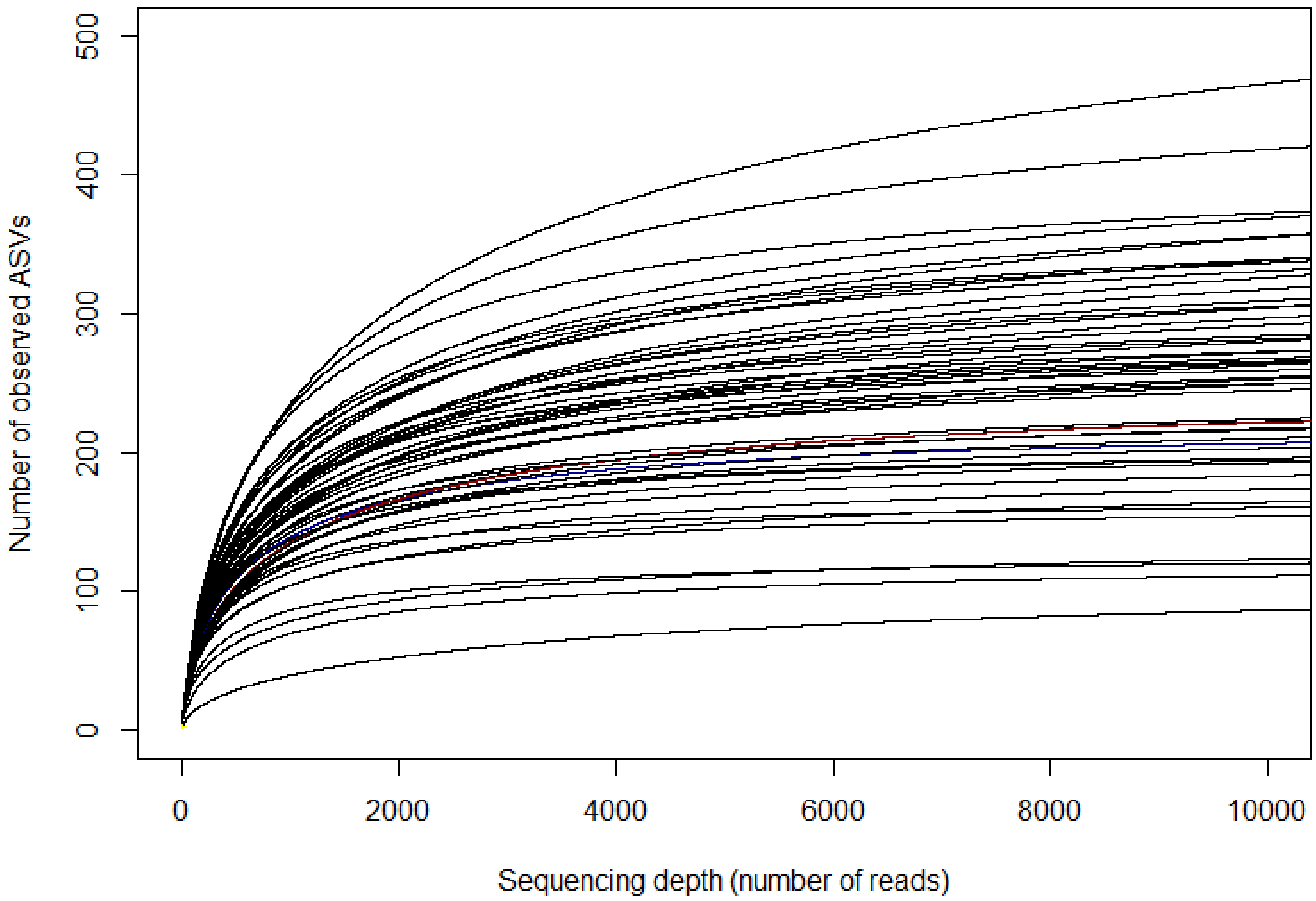

Figure S2. Rarefaction curves obtained from 16S rRNA sequencing for validation of the sequencing methodology. Black lines represent samples from cohort, blue line represents mock community, and red line represents mock community coupled with a stool sample. Rarefaction curves reached a plateau for all samples, therefore, the sequencing depth of the 16S rRNA gene amplicons was sufficient and our sequencing method was robust enough for subsequent bioinformatics analyses.

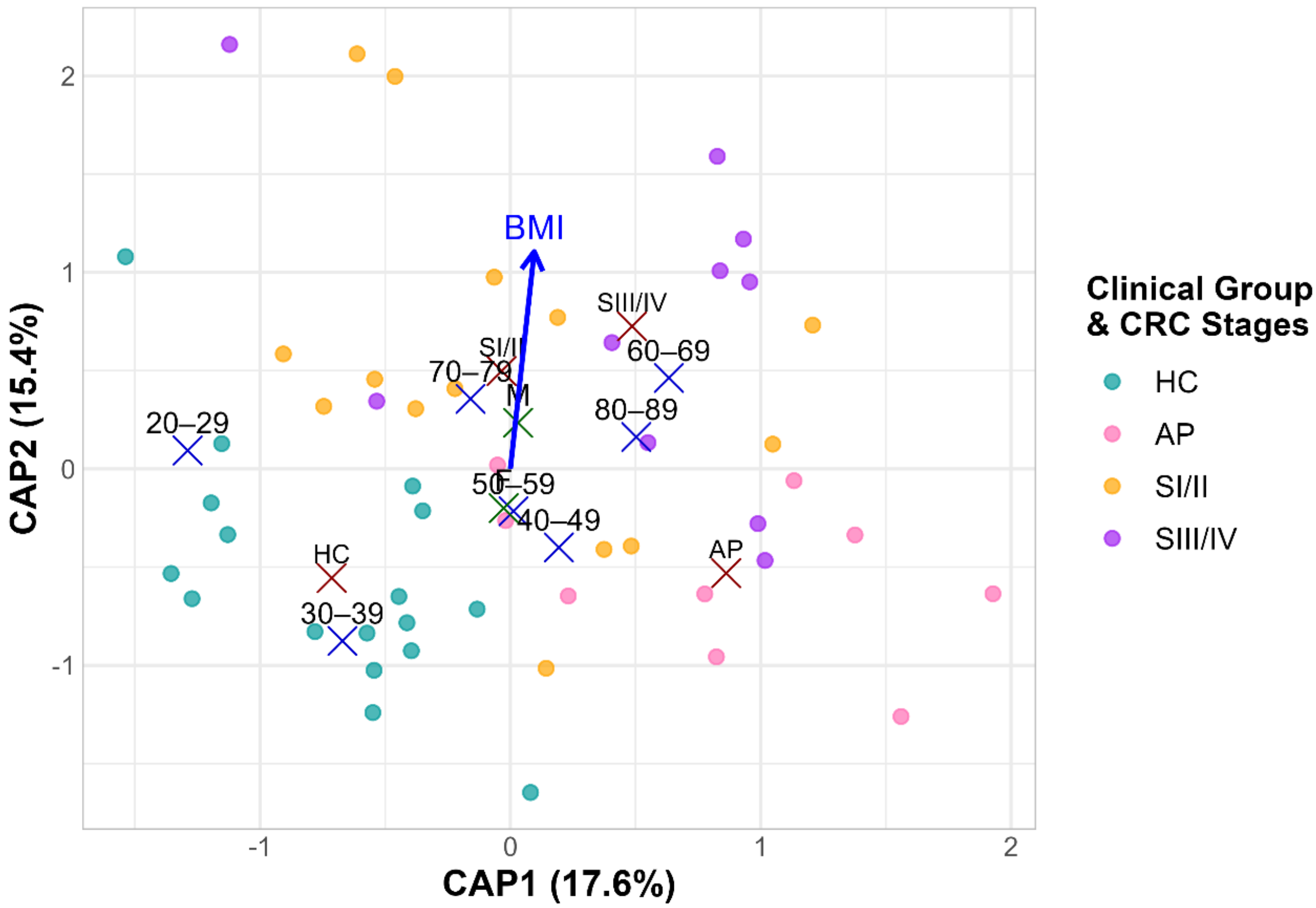

Figure S3. Distance-based redundancy analysis (dbRDA) of ASV-level Bray-Curtis dissimilarities, constrained by clinical group and colorectal cancer (CRC) stages, age group, sex, and BMI. Each point represents an individual sample and is colored according to clinical group/CRC stage. Cross-shaped markers indicate centroids for categorical variables (clinical group & CRC stages in red, age group in blue, sex in green), representing the mean ordination position of samples within each category. The blue arrow represents the direction and magnitude of BMI (continuous variable); arrow orientation indicates the direction of increasing BMI values. AP: adenoma patients; HC: healthy controls; CRC: colorectal cancer patients at stages I/II (SI/II) or I/II (SIII/IV); BMI: body mass index.

	AP vs. HC	SI/II vs. HC	SIII/IV vs. HC	SI/II vs. AP	SIII/IV vs. AP	SIII/IV vs. SI/II
Sutterella	5.01**	5.81**	6.68**	0.8	1.67	0.87
Terrisporobacter	-4.19**	-2.22**	-3.2**	1.97**	0.99	-0.99
Dialister	-0.58	1.05	2.83**	1.63*	3.41**	1.78*
Parasutterella	-2.43**	-1.76*	-3.13**	0.66	-0.7	-1.36
Lactobacillus	-2.88**	-3.01**	-3.02**	-0.13	-0.14	-0.01
Peptococcus	1.63*	2.79**	2.14*	1.15	0.51	-0.65
Howardella	-2.14**	-1.91**	-2.45**	0.23	-0.3	-0.54
Clostridia_UCG-014	-0.96	-1.68**	0.73	-0.72	1.69*	2.41**
[Eubacterium]_ruminantium_group	1.63**	0.71	-0.5	-0.92*	-2.13*	-1.21
Clostridium_sensu_stricto_1	-0.46	-0.84	-2.07*	-0.38	-1.61*	-1.23
Lachnospiraceae_UCG-010	0.7	1.1	1.98*	0.4	1.27*	0.87
UCG-010	-1.4	0.2	0.46	1.6*	1.86*	0.26
Slackia	0.11	-1.74**	-1.72**	-1.85**	-1.83**	0.02
[Eubacterium]_siraeum_group	-0.66	-1.84*	-0.61	-1.18	0.05	1.23
Barnesiella	-0.82	-0.02	-1.83*	0.8	-1.01	-1.81*
CAG-352	1.38*	0.89	1.82*	-0.49	0.44	0.93
Haemophilus	0.76	-1	-0.41	-1.76*	-1.17	0.59
Turicibacter	0.65	-0.73	0.99	-1.38**	0.34	1.72*
UCG-005	-1.31*	-1.63*	-0.28	-0.32	1.03	1.35
Coprococcus	1.6*	0.26	0.14	-1.35*	-1.46*	-0.11
Candidatus_Soleaferrea	0.95*	-0.53	-0.27	-1.48**	-1.21	0.26
NK4A214_group	-1.4*	-1.25*	-0.85	0.15	0.55	0.4
Desulfovibrio	0.72	0.43	1.38*	-0.29	0.66	0.96
Roseburia	0.03	-1.35*	-0.63	-1.38*	-0.66	0.72
Bilophila	-1.22*	-1.31*	-1.07	-0.09	0.15	0.24
UCG-003	-0.23	-0.46	0.84	-0.23	1.07	1.3*
Coprobacter	-0.45	-1.28*	-0.97	-0.83	-0.52	0.32
Phocea	-1.08*	0.1	0.02	1.18*	1.1	-0.09
UBA1819	0.09	1.14*	1.18	1.04	1.09	0.04
GCA-900066575	1.14**	0.19	0.95	-0.95*	-0.19	0.75
Lachnospiraceae_NK4A136_group	-1.05*	-1.09*	-0.28	-0.04	0.77	0.81
Holdemanella	0.55	0.71*	0.62	0.17	0.08	-0.09
Defluviitaleaceae_UCG-011	0	-0.67*	-0.52	-0.67	-0.53	0.15

Color scale: 8, 4, 0, -4, -8

Figure S4. Heatmap of the multiple pairwise comparison analysis output from ANCOM-BC2, evaluating differentially abundant bacterial genera between all clinical groups (HC, AP, and colorectal cancer (CRC) stages SI/II and SIII/IV). Each cell represents a log fold-change (LFC, natural log) value of the corresponding comparison. Cells are color-coded, red represents increased abundance and blue represents decreased abundance. LFC values followed by an asterisk (*) represent significant changes without adjustment for multiple testing correction ($p < 0.05$), whereas values followed by two asterisks (**) are statistically significant after adjustment for mixed directional false discovery rate (Benjamini-Hochberg method, $q < 0.05$). AP: adenoma patients; HC: healthy controls; CRC: colorectal cancer patients at stages I/II (SI/II) or I/II (SIII/IV).

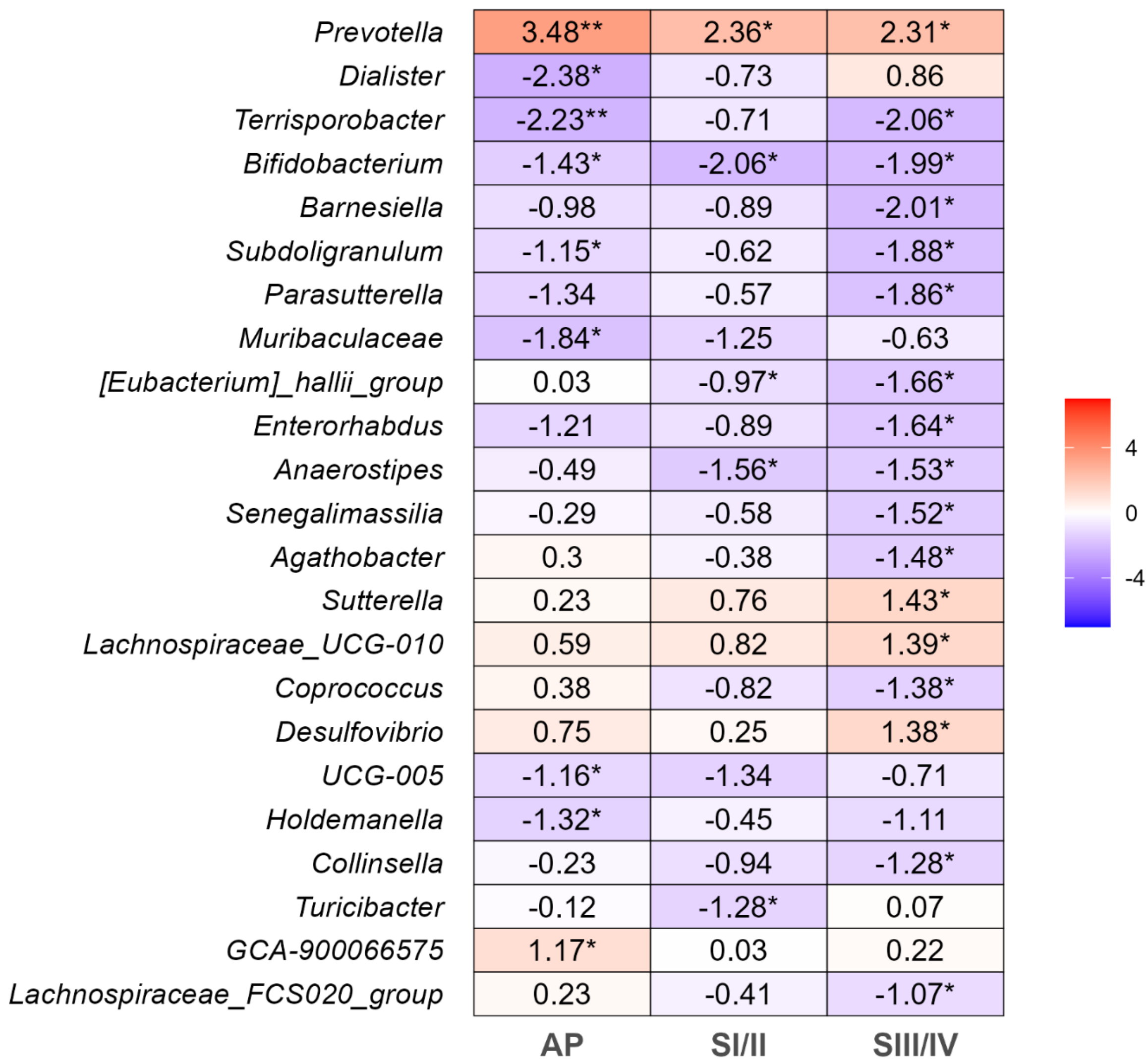

Figure S5. Results of ANCOM-BC2 analysis model without covariate adjustment. Values represent log fold-change values (LFC, natural log) of taxa abundance in AP and CRC stages (SI/II and SIII/IV) comparatively to HC (reference group). Cells are color-coded on a gradient, where red indicates increased abundance and purple/blue indicates decreased abundance. LFC values followed by an asterisk (*) represent significant changes without adjustment for multiple testing correction ($p < 0.05$), whereas values followed by two asterisks (**) are statistically significant after adjustment for mixed directional false discovery rate (Benjamini-Hochberg method, $q < 0.05$). HC: healthy controls; AP: adenoma patients; CRC: colorectal cancer patients.

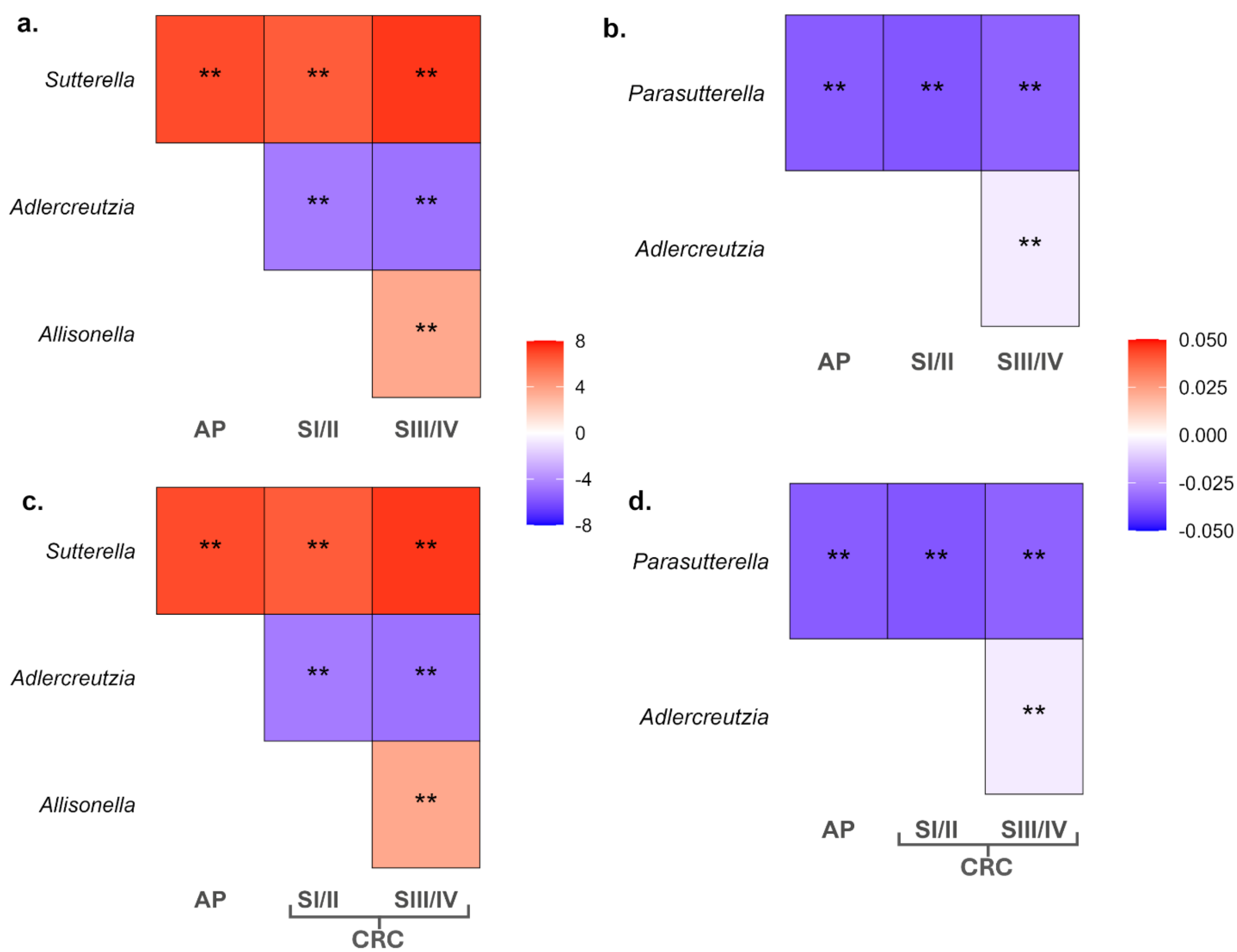

Figure S6. Heatmap showing microbial features significantly associated with clinical groups as determined by MaAsLin2, adjusted for covariates (age group, sex, BMI). Differential abundance (DA) analysis was performed for the original cohort (n = 50), respectively, **(a)** filtered by 10% prevalence prior to analysis; **(c)** unfiltered; plus, sensitivity analysis on BMI-matched cohort (n = 26) **(b)** filtered by 10% prevalence, and **(d)** unfiltered. MaAsLin2 analyses performed for age-matched and sex-matched cohorts resulted in no significantly altered taxa. Color scale indicates the strength of association (MaAsLin2 model coefficient, effect size), with red representing positive associations (higher relative abundance) and blue representing negative associations (lower relative abundance) as compared to the reference group (HC). Only features meeting the standard MaAsLin2 significance threshold (false discovery rate-adjusted, Benjamini-Hochberg method, $q \leq 0.25$) in at least one comparison are displayed (**). HC: healthy controls; AP: adenoma patients; CRC: colorectal cancer patients at stages I/II (SI/II) or III/IV (SIII/IV); BMI: body mass index.

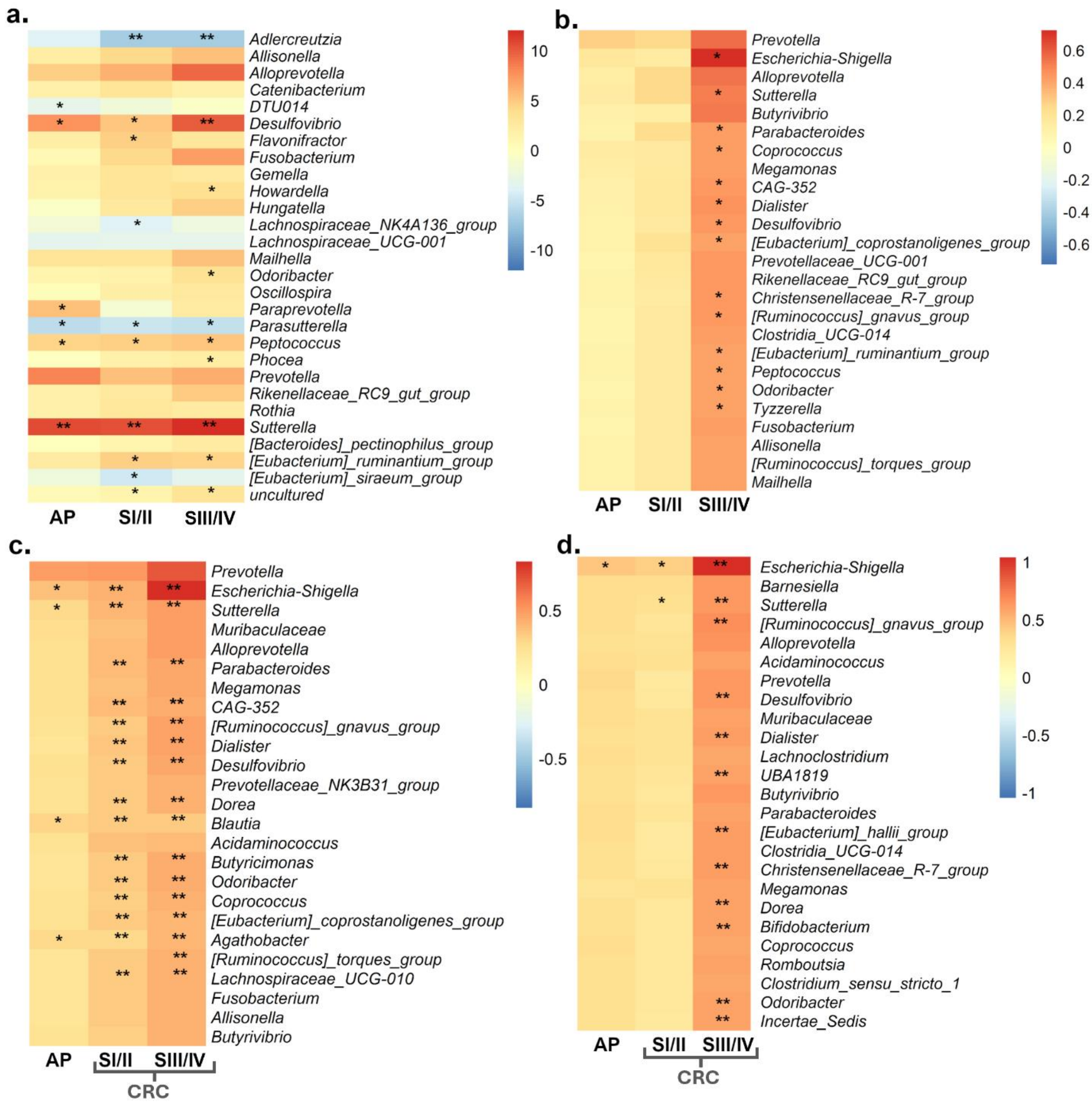

Figure S7. Heatmap of limma voom differential abundance (DA) analysis results, showing contrasts in $\log_2$ fold change of genus abundance across clinical groups, as compared to HC (reference), adjusted for age group, sex, and BMI. Taxa were filtered by prevalence prior to analysis (>10%). **(a)** original cohort (n = 50); **(b)** age-matched cohort (n = 25); **(c)** sex-matched cohort (n = 36); **(d)** BMI-matched cohort (n = 26). Genera displayed in each heatmap correspond to the top 25 features from each corresponding output, by $\log_2$ fold change values. Color scale indicates direction and magnitude of differential abundance (red: increased; blue: decreased relative to HC). Statistically significant genera are represented by an asterisk (*); whereas two asterisks (**) represent genera significantly altered after multiple testing adjustment ($q < 0.05$, Benjamini-Hochberg method). HC: healthy controls; AP:

adenoma patients; CRC: colorectal cancer patients at stages I/II (SI/II) or III/IV (SIII/IV); BMI: body mass index.

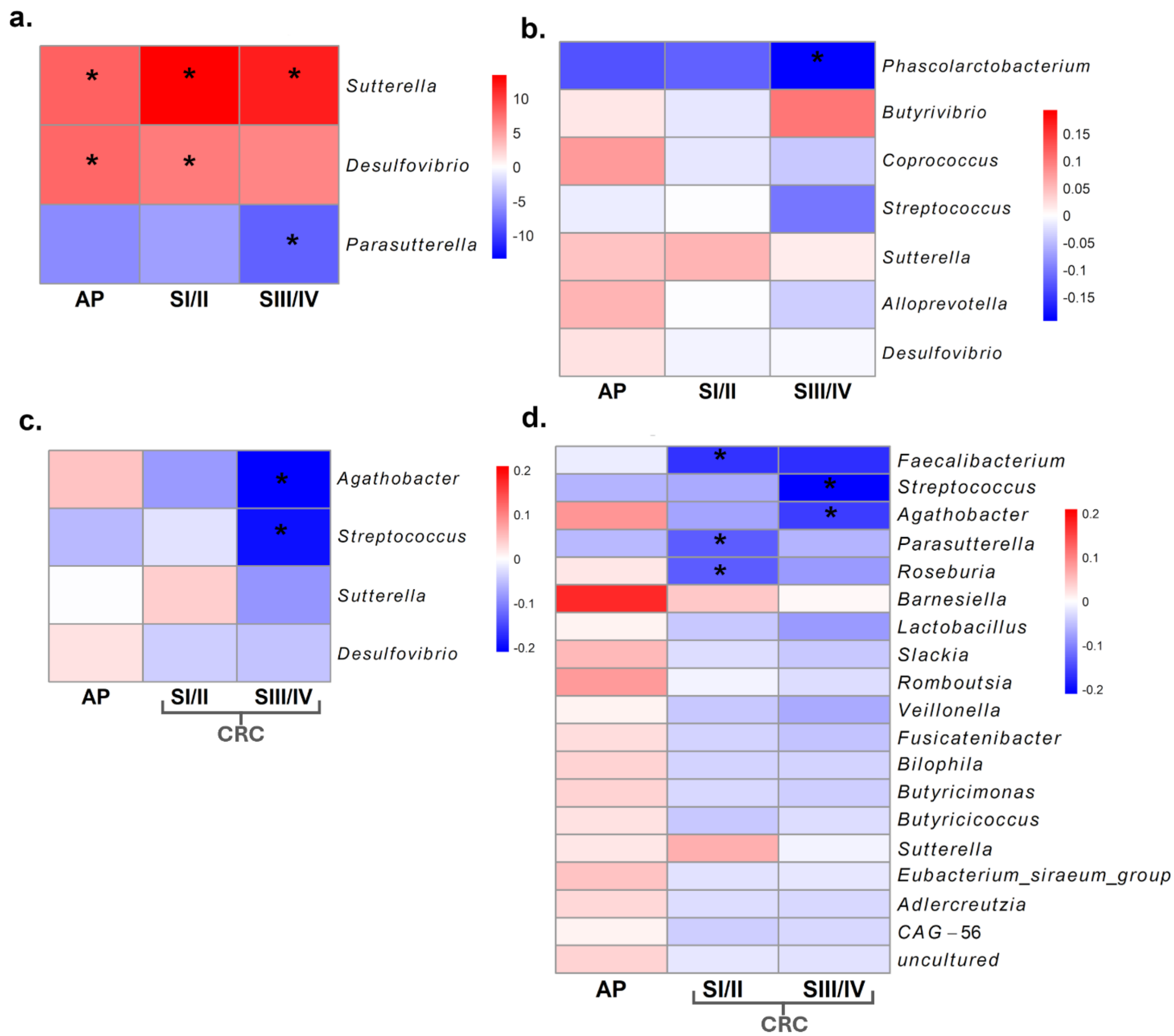

Figure S8. Heatmaps showing $\log_2$ fold change differences in relative abundance of bacterial genera across clinical groups, compared to HC (reference), as calculated by fastANCOM, adjusting for age group, sex, and BMI covariates. Taxa were filtered by prevalence prior to analysis (>10%). Heatmaps represent fastANCOM outputs for: **(a)** original cohort (n = 50); **(b)** age-matched cohort (n = 25); **(c)** sex-matched cohort (n = 36); **(d)** BMI-matched cohort (n = 26). Color scale indicates enrichment (red) or depletion (blue) relative to HC. Genera identified as differentially abundant by fastANCOM (REJECT = TRUE) and that are statistically significant are represented by an asterisk (*); whereas differentially abundant genera statistically significant after multiple testing adjustment (q < 0.05, Benjamini-Hochberg correction) are represented by two asterisks (**). HC: healthy controls; AP: adenoma patients; CRC: colorectal cancer patients at stages I/II (SI/II) or III/IV (SIII/IV).

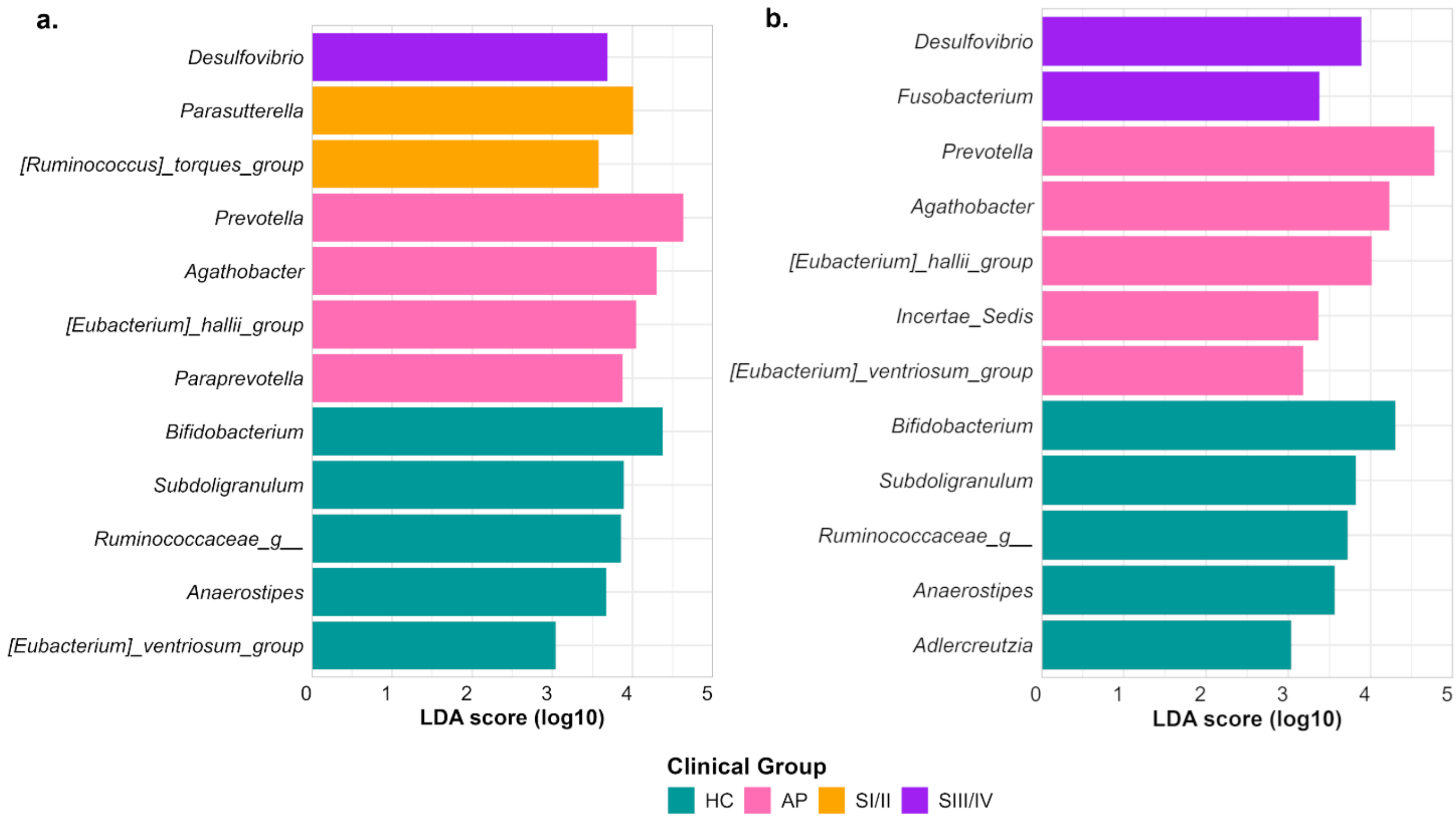

Figure S9. Bar plots of significantly enriched genera according to LEfSe analysis (LDA score > 3, $p < 0.05$), by clinical group. **(a)** Data filtered by prevalence (10%); **(b)** Unfiltered data. LDA: linear discriminant analysis; HC: healthy controls; AP: adenoma patients; CRC: colorectal cancer patients at stages I/II (SI/II) or III/IV (SIII/IV).

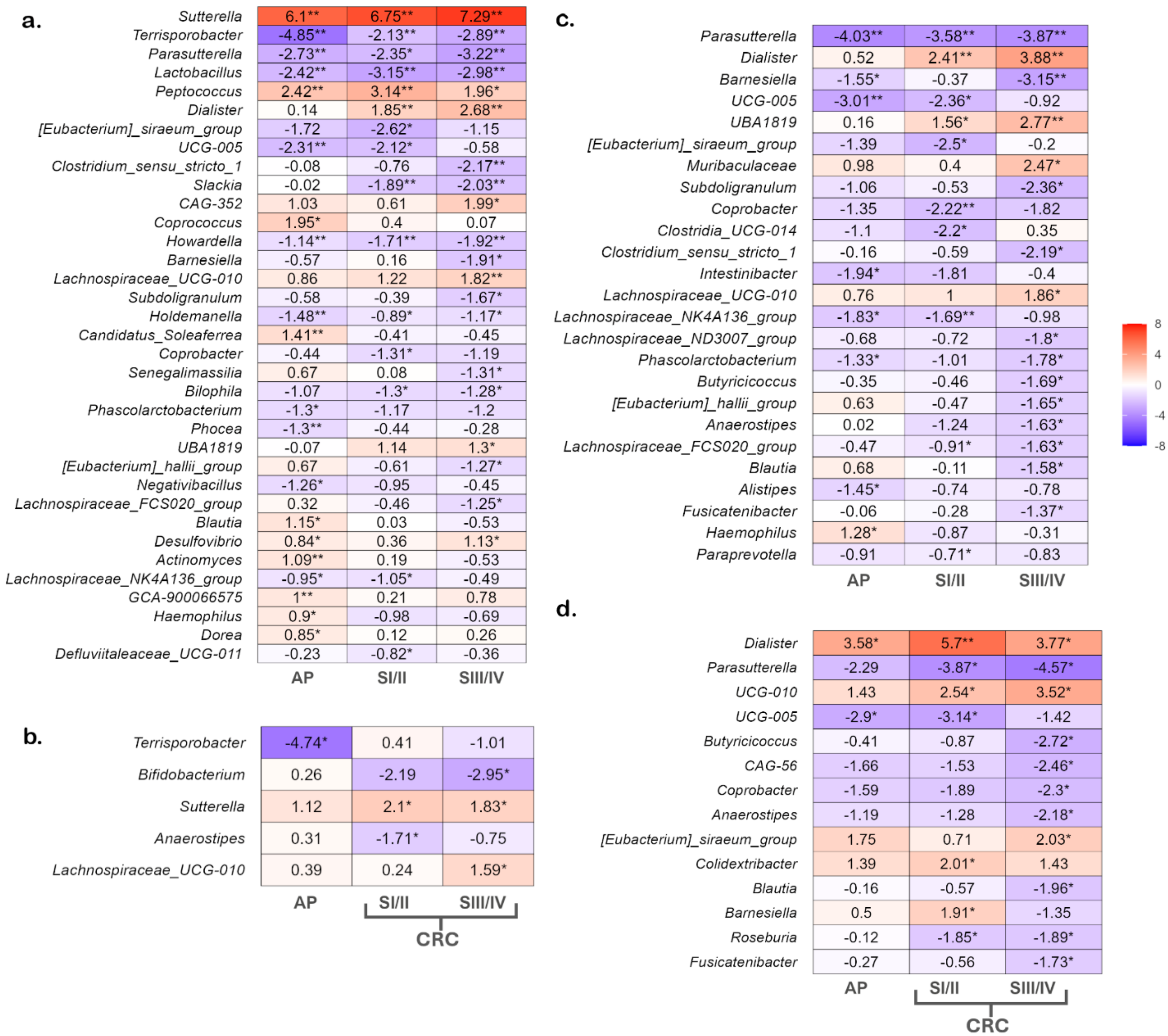

Figure S10. Sensitivity analysis by ANCOM-BC2 global analysis (adjusted for covariates: age group, sex, BMI), evaluating differential abundance of bacterial genera. No prevalence filtering was applied prior to this analysis. **(a)** Original cohort (*n* = 50); **(b)** age-matched cohort (*n* = 25); **(c)** sex-matched cohort (*n* = 36); **(d)** BMI-matched cohort (*n* = 26). The numbers represent log fold-change values (LFC, natural log) of taxa abundance in AP and CRC stages (SI/II and SIII/IV) comparatively to HC (reference group). Cells are color-coded on a gradient, where red indicates increased abundance and purple/blue indicates decreased abundance. LFC values followed by an asterisk (*) represent significant changes without adjustment for multiple testing correction ($p < 0.05$), whereas values followed by two asterisks (**) are statistically significant after adjustment for mixed directional false

discovery rate (Benjamini-Hochberg method, $q < 0.05$). HC: healthy controls; AP: adenoma patients; CRC: colorectal cancer patients at stages I/II (SI/II) or I/II (SIII/IV); BMI: body mass index.

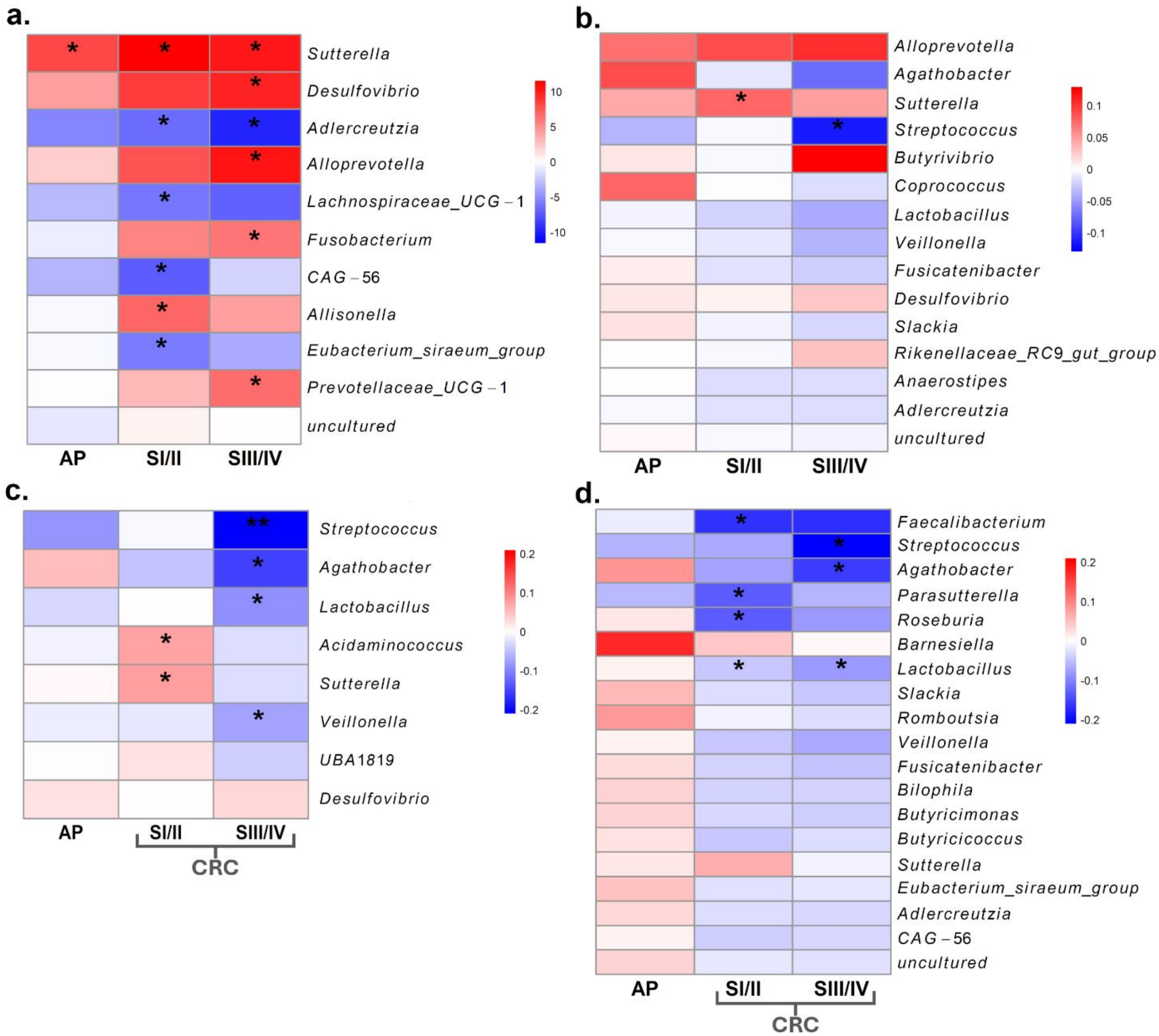

Figure S11. Heatmaps showing differences in $\log_2$ fold change of relative abundance of bacterial genera across clinical groups, as compared to HC (reference), as calculated by fastANCOM, adjusting for age group, sex, and BMI covariates. Taxa were not filtered by prevalence prior to analysis. Heatmaps represent fastANCOM outputs for: **(a)** original cohort (n = 50); **(b)** age-matched cohort (n = 25); **(c)** sex-matched cohort (n = 36); **(d)** BMI-matched cohort (n = 26). Color scale indicates enrichment (red) or depletion (blue) relative to HC. Genera identified as differentially abundant by fastANCOM (REJECT = TRUE) and that are statistically significant are represented by an asterisk (*); whereas differentially abundant genera statistically significant after multiple testing adjustment (q <

0.05, Benjamini-Hochberg correction) are represented by two asterisks (**). HC: healthy controls; AP: adenoma patients; CRC: colorectal cancer patients at stages I/II (SI/II) or I/II (SIII/IV); BMI: body mass index.

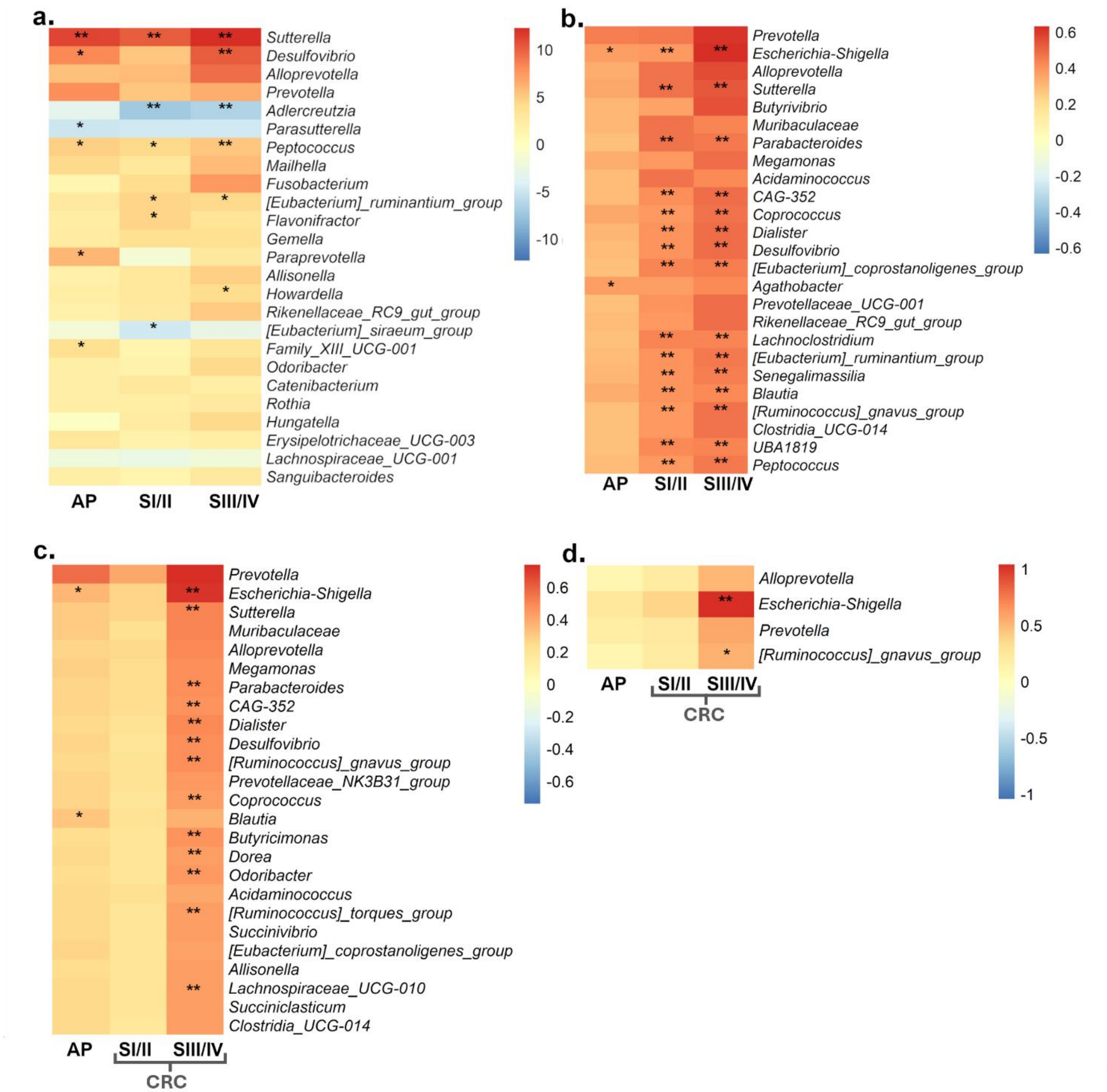

Figure S12. Heatmap of limma voom differential abundance (DA) analysis results, showing contrasts in $\log_2$ fold change of genus abundance across clinical groups, as compared to HC (reference), adjusted for age group, sex, and BMI. Taxa were not filtered by prevalence prior to analysis. **(a)** original cohort

(*n* = 50); **(b)** age-matched cohort (*n* = 25); **(c)** sex-matched cohort (*n* = 36); **(d)** BMI-matched cohort (*n* = 26). Genera displayed in each heatmap correspond to the top 25 features from each corresponding output, by log_2 fold change values. Color scale indicates direction and magnitude of differential abundance (red: increased; blue: decreased relative to HC). Statistically significant genera are represented by an asterisk (*); whereas two asterisks (**) represent genera significantly altered after multiple testing adjustment ($q < 0.05$, Benjamini-Hochberg method). HC: healthy controls; AP: adenoma patients; CRC: colorectal cancer patients at stages I/II (SI/II) or I/II (SIII/IV); BMI: body mass index.

a.

	AP	SI/II	SIII/IV
Sutterella	5.01**	5.81**	6.68**
Terrisporobacter	-4.19**	-2.22**	-3.2**
Parasutterella	-2.43**	-1.76*	-3.13**
Lactobacillus	-2.88**	-3.01**	-3.02**
Dialister	-0.58	1.05	2.83**
Peptococcus	1.63*	2.79**	2.14*
Howardella	-2.14**	-1.91**	-2.45**
Clostridium_sensu_stricto_1	-0.46	-0.84	-2.07*
Lachnospiraceae_UCG-010	0.7	1.1	1.98*
[Eubacterium]_siraeum_group	-0.66	-1.84*	-0.61
Barnesiella	-0.82	-0.02	-1.83*
CAG-352	1.38*	0.89	1.82*
Slackia	0.11	-1.74**	-1.72*
Clostridia_UCG-014	-0.96	-1.68*	0.73
UCG-005	-1.31*	-1.63*	-0.28
[Eubacterium]_ruminantium_group	1.63**	0.71	-0.5
Coprococcus	1.6*	0.26	0.14
NK4A214_group	-1.4*	-1.25*	-0.85
Desulfovibrio	0.72	0.43	1.38*
Roseburia	0.03	-1.35*	-0.63
Bilophila	-1.22*	-1.31*	-1.07
Coprobacter	-0.45	-1.28*	-0.97
UBA1819	0.09	1.14*	1.18
GCA-900066575	1.14**	0.19	0.95
Lachnospiraceae_NK4A136_group	-1.05*	-1.09*	-0.28
Phocea	-1.08*	0.1	0.02
Candidatus_Soleaferrea	0.95*	-0.53	-0.27
Holdemanella	0.55	0.71*	0.62
Defluviitaleaceae_UCG-011	0	-0.67*	-0.52

b.

	AP	SI/II (CRC)	SIII/IV (CRC)
Terrisporobacter	-4.79*	0.26	-0.92
Bifidobacterium	0.28	-2.28	-2.86*
CAG-352	1.63	0.67	2.2*
Sutterella	1.11	2.03*	1.92*
Anaerostipes	0.31	-1.78*	-0.68
Lachnospiraceae_UCG-010	0.38	0.22	1.68*

c.

	AP	SI/II	SIII/IV
Parasutterella	-4.02**	-3.57**	-3.87**
Dialister	0.53	2.46**	3.9**
Barnesiella	-1.56	-0.32	-3.16**
UCG-005	-3.03**	-2.38*	-0.87
UBA1819	0.18	1.57*	2.8**
Muribaculaceae	0.99	0.45	2.59*
[Eubacterium]_siraeum_group	-1.36	-2.42*	-0.15
Subdoligranulum	-1.01	-0.51	-2.29*
Clostridia_UCG-014	-1.1	-2.18*	0.38
Coprobacter	-1.32	-2.15**	-1.81
Clostridium_sensu_stricto_1	-0.13	-0.54	-2.13*
Intestinibacter	-1.94*	-1.8	-0.34
Lachnospiraceae_UCG-010	0.79	1.08	1.91*
Lachnospiraceae_NK4A136_group	-1.82*	-1.63**	-0.94
Lachnospiraceae_ND3007_group	-0.73	-0.69	-1.75*
Butyricicoccus	-0.35	-0.41	-1.62*
[Eubacterium]_hallii_group	0.65	-0.42	-1.61*
Anaerostipes	0.02	-1.22	-1.6*
Lachnospiraceae_FCS020_group	-0.52	-0.88	-1.58*
Blautia	0.7	-0.08	-1.52*
Haemophilus	1.35*	-0.78	-0.21
Fusicatenibacter	-0.07	-0.26	-1.34*

Color scale: 8, 4, 0, -4, -8

d.

	AP	SI/II (CRC)	SIII/IV (CRC)
Dialister	3.8*	5.94**	3.87*
Parasutterella	-2.31	-3.94*	-4.69*
UCG-010	1.44	2.54*	3.41*
UCG-005	-3*	-3.31*	-1.5
Butyricicoccus	-0.39	-0.85	-2.7*
CAG-56	-1.56	-1.42	-2.52*
Coprobacter	-1.56	-1.82	-2.38*
Anaerostipes	-1.23	-1.33	-2.23*
Colidextribacter	1.38	2.02*	1.33
Barnesiella	0.55	1.98*	-1.36
Roseburia	-0.09	-1.81	-1.97*
Blautia	-0.17	-0.62	-1.96*
[Eubacterium]_siraeum_group	1.8	0.77	1.96*
Fusicatenibacter	-0.25	-0.51	-1.81*
[Ruminococcus]_gauvreauii_group	-0.06	-1.04	-1.71*

Figure S13. Sensitivity analysis by ANCOM-BC2 global analysis (adjusted for covariates: age group, sex, BMI), evaluating differential abundance of bacterial genera. Taxa were filtered by a minimum threshold of 10% prevalence. **(a)** original cohort (n = 50); **(b)** age-matched cohort (n = 25); **(c)** sex-matched cohort (n = 36); **(d)** BMI-matched cohort (n = 26). The numbers represent log fold-change values (LFC, natural log) of taxa abundance in AP and CRC stages (SI/II and SIII/IV) comparatively to HC (reference group). Cells are color-coded on a gradient, where red indicates increased abundance and purple/blue indicates decreased abundance. LFC values followed by an asterisk (*) represent significant changes without adjustment for multiple testing correction ($p < 0.05$), whereas values followed by two asterisks (**) are statistically significant after adjustment for mixed directional false

discovery rate (Benjamini-Hochberg false discovery rate (FDR) correction, $q < 0.05$). HC: healthy controls; AP: adenoma patients; CRC: colorectal cancer patients at stages I/II (SI/II) or I/II (SIII/IV); BMI: body mass index.

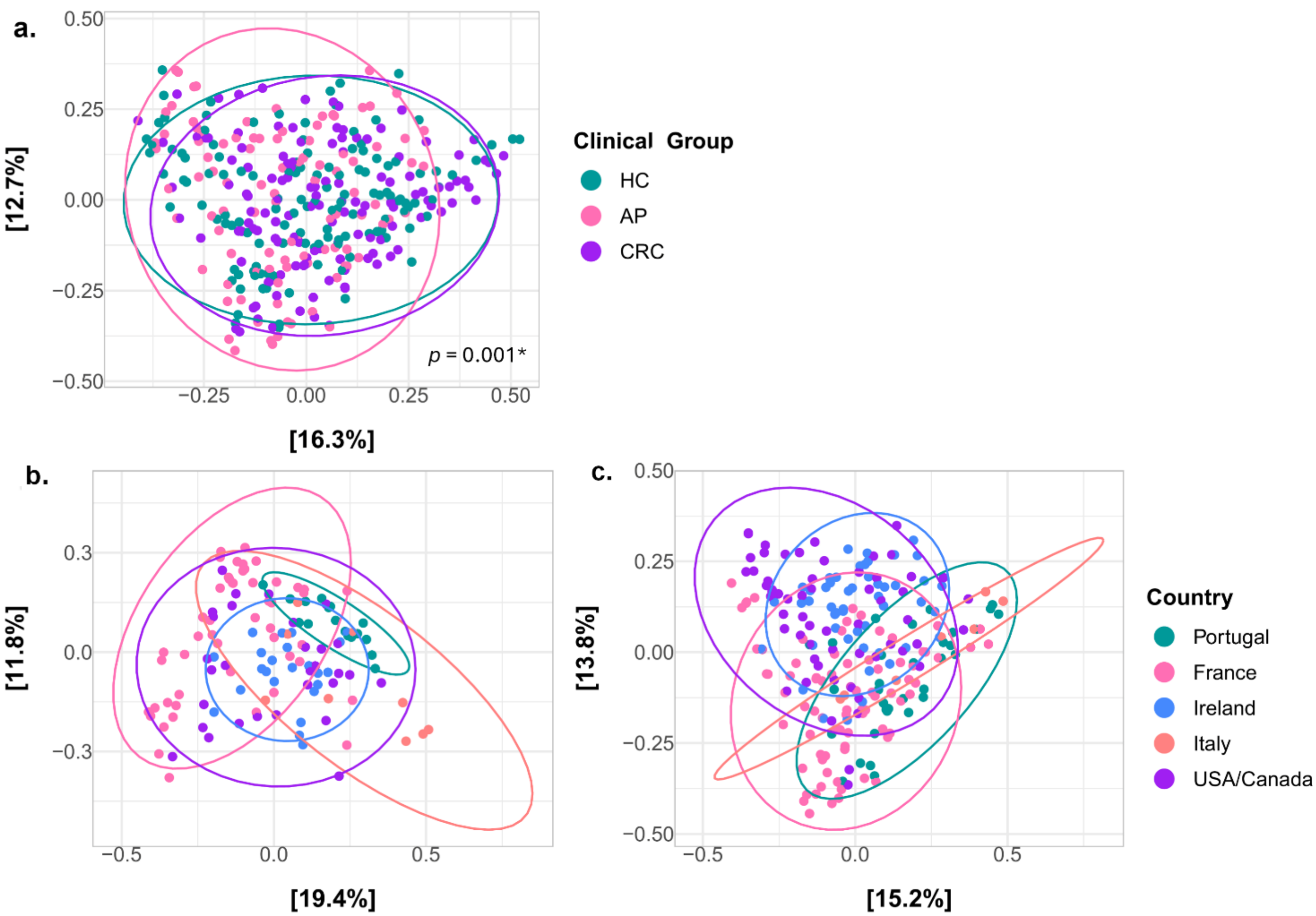

Figure S14. Microbial community composition of the pooled cohorts from Portugal, France, Ireland, Italy, and USA/Canada. Beta diversity was calculated through Bray-Curtis dissimilarity, represented by PCoA plot: **(a)** all cohorts by clinical group. **(b)** healthy subjects from all cohorts plotted by country of origin; **(c)** diseased patients (AP and CRC patients) from all cohorts by country of origin. HC: healthy controls; AP: adenoma patients; CRC: colorectal cancer patients.
